# LC–MS/MS-based phospholipid profiling of plant-pathogenic bacteria with tailored separation of methyl-branched species

**DOI:** 10.1007/s00216-024-05451-1

**Published:** 2024-07-25

**Authors:** Edward Rudt, Christian Faist, Vera Schwantes, Nele Konrad, Nina Wiedmaier-Czerny, Katja Lehnert, Shiri Topman-Rakover, Aya Brill, Saul Burdman, Zvi Hayouka, Walter Vetter, Heiko Hayen

**Affiliations:** 1 Institute of Inorganic and Analytical Chemistry, University of Münster, Corrensstraße 48, D-48149 Münster, Germany; 2 Institute of Food Chemistry (170b) , University of Hohenheim, Garbenstraße 28, D-70593 Stuttgart, Germany; 3 Institute of Biochemistry, Food Science and Nutrition, The Robert H. Smith Faculty of Agriculture, Food & Environment, The Hebrew University of Jerusalem, 76100 Rehovot, Israel; 4 Department of Plant Pathology and Microbiology, The Robert H. Smith Faculty of Agriculture, Food & Environment, The Hebrew University of Jerusalem, 76100 Rehovot, Israel

**Keywords:** Phospholipid, Lipidomics, Branched-chain fatty acid, Plant-pathogenic bacteria, LC–MS/MS, GC/MS

## Abstract

Plant-pathogenic bacteria are one of the major constraints on agricultural yield. In order to selectively treat these bacteria, it is essential to understand the molecular structure of their cell membrane. Previous studies have focused on analyzing hydrolyzed fatty acids (FA) due to the complexity of bacterial membrane lipids. These studies have highlighted the occurrence of branched-chain fatty acids (BCFA) alongside normal-chain fatty acids (NCFA) in many bacteria. As several FA are bound in the intact phospholipids of the bacterial membrane, the presence of isomeric FA complicates lipid analysis. Furthermore, commercially available reference standards do not fully cover potential lipid isomers. To address this issue, we have developed a reversed-phase high-performance liquid chromatography (RP-HPLC) method with tandem mass spectrometry (MS/MS) to analyze the phospholipids of various plant-pathogenic bacteria with a focus on BCFA containing phospholipids. The study revealed the separation of three isomeric phosphatidylethanolamines (PE) depending on the number of bound BCFA to NCFA. The validation of the retention order was based on available reference standards in combination with the analysis of hydrolyzed fatty acids through gas chromatography with mass spectrometry (GC/MS) after fractionation. Additionally, the transferability of the retention order to other major lipid classes, such as phosphatidylglycerols (PG) and cardiolipins (CL), was thoroughly examined. Using the information regarding the retention behavior, the phospholipid profile of six plant-pathogenic bacteria was structurally elucidated. Furthermore, the developed LC–MS/MS method was used to classify the plant-pathogenic bacteria based on the number of bound BCFA in the phospholipidome.

**Graphical Abstract:**

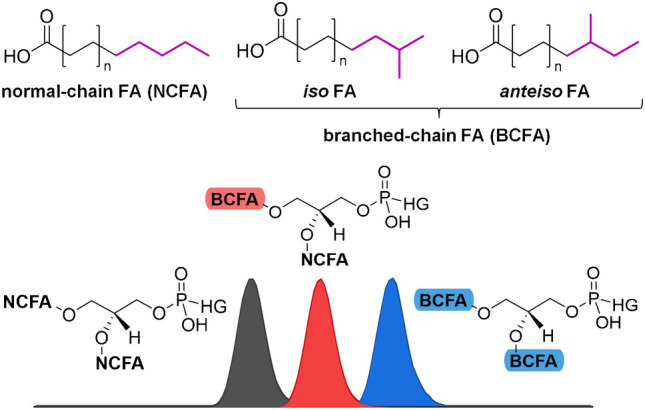

**Supplementary Information:**

The online version contains supplementary material available at 10.1007/s00216-024-05451-1.

## Introduction

Plant-pathogenic bacteria pose a major problem for food production efficiency as they infest a wide variety of plants and fruits in agricultural fields, leading to a significant reduction in crop yield [1]. Examples of plant-pathogenic bacterial diseases include potato scab and bacterial spot disease of tomato caused by *Streptomyces scabies* (*Ssc*) [2, 3] and *Pseudomonas syringae* pv. *tomato* (*Pst*) [4, 5], respectively. To enhance food production efficiency, it has become necessary to use selective crop protection products for treatment [6, 7]. However, the development of selective agents requires knowledge of the biological composition of distinct bacteria [8]. In their study, Wiedmaier-Czerny et al. [9] revealed the hydrolyzed FA pattern for major plant-pathogenic bacteria [10], including Gram-positive *Clavibacter michiganensis* (*Cm*, formerly known as *Clavibacter michiganensis* subsp. *michiganensis*) and *Ssc*, as well as Gram-negative *Paracidovorax citrulli* (*Pc*, formerly known as *Acidovorax citrulli*), *Pst*, *Xanthomonas campestris* pv. *campestris* (*Xcc*), and *Xanthomonas euvesicatoria* pv. *perforans* (*Xep*, formerly known as *Xanthomonas perforans*). Next to saturated FA and monounsaturated fatty acids (MUFA), BCFA have been proven to be present in *Cm*, *Ssc*, *Xcc*, and *Xep* [9]. These BCFA are further classified into *iso*-fatty acids (*i*FA, branching at the penultimate carbon) and *anteiso*-fatty acids (*a*FA, branching at the antepenultimate carbon) [11]. In addition to MUFA, BCFA serve to improve membrane fluidity and stability [12, 13]. While *i*FA are built from valine and leucine (even- or odd-numbered species, respectively), *a*FA are solely based on isoleucine (odd-numbered species) [11].

Despite the results on hydrolyzed fatty acids, the intact phospholipid composition of the bacterial membranes remains an area of interest to achieve further understanding of the bacteria [14]. Due to the combination of several potentially isomeric fatty acids bound, developing suitable analytical methods poses a considerable challenge [15, 16]. Therefore, some previous studies on bacterial phospholipids did not differentiate between BCFA and NCFA containing lipids [17, 18]. In addition, method development for bound BCFA analysis in lipids is complicated due to the limited number of suitable phospholipid reference standards, unlike for free BCFA [19]. To elucidate isomeric FA, gas chromatography/mass spectrometry (GC/MS) is commonly used due to its high chromatographic resolving power [20, 21]. GC/MS additionally brings the advantage of producing diagnostic fragment ions for structural elucidation by breaking C–C bonds through harsh electron ionization (EI) [22]. However, this technique is not feasible for phospholipid analysis due to their thermal decomposition in the GC.

In lipidomics, LC–MS is commonly used for comprehensive characterization of complex samples including isomeric lipid species [23, 24]. Hydrophilic interaction liquid chromatography (HILIC) is becoming increasingly popular for phospholipid analysis due to class-specific separation [25] and for that reason also for differentiation of isomeric lipid classes according to their varying head group polarity [25, 26]. For separating isomeric lipid species based on differences in the hydrophobic fatty acyl moieties, reversed-phase (RP)-HPLC remains the method of choice [19, 27]. RP-HPLC methods have also been developed to distinguish between BCFA and NCFA as an alternative to GC/MS [28]. Additionally, both GC/MS [29] and LC–MS [30, 31] can be used to perform enantioselective separations of BCFA with dedicated columns to separate enantiomeric *a*FA. However, unlike GC/MS, LC–MS/MS fragmentation experiments do not provide structural elucidation of BCFA, since C–C bonds are not cleaved by usual collision-induced dissociation (CID) [32]. To overcome this problem, various techniques have been developed to achieve radical-induced fragmentation of C–C bonds in lipidomics [33, 34]. However, these techniques have a drawback in the structural elucidation of BCFA containing lipids due to the low abundance of diagnostic fragment ions and the high susceptibility to errors in chimeric MS/MS spectra [19]. Therefore, chromatography must be used additionally to distinguish between BCFA and NCFA containing lipids [35].

Due to the high complexity of BCFA containing lipids and the lack of reference standards, there are only a few studies on the LC–MS analysis of esterified BCFA and NCFA in lipids. These studies focused either on the analysis of BCFA containing lyso compounds [36] or on the analysis of di-acylglycerols [37] and triacylglycerols [38] by using multiple analytical columns connected in series with long gradient elution. Recently, Freeman et al. [19] analyzed BCFA containing PG in *Staphylococcus aureus*. The study revealed the retention behavior of BCFA and NCFA containing lipids for diacylated lipids using stable isotope labelling. RP-HPLC was used to separate diacylated lipids based on the degree of esterified BCFA, resulting in the observation of three peaks (two BCFA containing PG, one BCFA containing PG, and none BCFA containing PG, respectively). However, unlike in some BCFA studies, no differentiation between *i*FA and *a*FA was achieved at intact phospholipid level [19].

Despite the results of Freeman et al. [19], further validation of this retention order is required. For instance, the transfer of this retention order to other lipid classes or lipid species featuring MUFA was not investigated.

Therefore, we developed an RP–HPLC–MS/MS method for phospholipid profiling of major plant-pathogenic bacteria with a focus on methyl-branched species. For this purpose, we reinvestigated six plant-pathogenic samples from the hydrolyzed fatty acid study of Wiedmaier-Czerny et al., including *Pc*, *Cm*, *Pst*, *Ssc*, *Xcc*, and *Xep*. Recently introduced commercially available reference standards and HPLC fractionation followed by GC/MS analysis of the hydrolyzed fatty acids were utilized to validate the retention order of BCFA and NCFA containing lipids. Using the developed RP–HPLC–MS/MS method, the phospholipid profile of the six plant-pathogenic bacteria was revealed and a detailed comparison of the lipid composition based on the number of bound BCFA was carried out for further characterization of these bacteria.

## Material and methods

### Chemicals

1-(12*S*-Methylmyristoyl)-2-(13-methylmyristoyl)-*sn*-glycero-3-phosphoethanolamine (PE *a*15:0/*i*15:0), 1,2-dipentadecanoyl-*sn*-glycero-3-phosphoethanolamine (PE 15:0/15:0), and ammonium acetate (NH_4_OAc, trace metal basis) were purchased from Sigma-Aldrich (Steinheim, Germany). 1-Palmitoyl-2-hydroxy-*sn*-glycero-3-phosphoethanolamine (LPE 16:0), 1,2-dihexadecanoyl-*sn*-glycero-3-phospho-(1′-*sn*-glycerol) (PG 16:0/16:0), and 18:2 cardiolipin (CL 72:8) were obtained from Biomol (Hamburg, Germany). Pentadecanoic acid was from Merck (Darmstadt, Germany); *iso-* and *anteiso*-fatty acid standards were purchased from Larodan (Malmö, Sweden). LC–MS grade acetonitrile (ACN), 2-propanol (IPA), and methanol (MeOH) as well as glacial acetic acid (AA, ACS Reag. Ph. Eur.) were acquired from VWR International (Darmstadt, Germany). LC grade methyl-*tert*-butyl ether (MTBE) was obtained from Merck (Darmstadt, Germany). HPLC grade *n*-hexane was purchased from Th. Geyer (Renningen, Germany). Sulfuric acid (96% purity) was acquired from Carl Roth (Karlsruhe, Germany). Butylhydroxytoluol (BHT) was obtained from Fisher Scientific (Schwerte, Germany). Purified water from a Milli-Q EQ 7000 system from Merck (Darmstadt, Germany) was utilized.

### Cultivation

Freeze-dried bacterial strains, used in the previous fatty acid study by Wiedmaier-Czerny et al. [9], were reinvestigated for intact phospholipid profiling. The following strains were cultivated using the standard protocol of Topman et al. [8]: *Pc*, *Cm*, *Pst*, *Ssc*, *Xcc*, and *Xep*. In brief, four batches of bacteria in NB medium (Difco, 50 mL each) were incubated overnight in a shaker (180 rpm, 28 °C). The combined batches were then diluted with NB medium to an OD of 0.1 (600 nm). Aliquots of 500 mL were used for further preparation. The suspensions were incubated again (24 h, 180 rpm, 28 °C). After centrifugation (8000 rpm, 15 min), the supernatant was discarded, and the bacterial pellets were washed 3 × with PBS and 2 × with H_2_O, subsequently. The cultivated bacteria were then dissolved in H_2_O and freeze-dried for further sample preparation.

### Lipid extraction

The freeze-dried bacteria underwent lipid extraction following the protocol described by Matyash et al. [39]. Ten milligrams of each bacterial strain (two biological replicates) was dissolved in 1.3 mL MeOH and 5 mL MTBE. In addition, 10 µL CL 72:8 (100 µM) was added as an extraction marker, along with 200 µL BHT (50 mM in MeOH) as an antioxidant. After incubation for 1 h at room temperature, 1.25 mL H_2_O was added, and the bacteria were further incubated for 10 min. The samples underwent centrifugation at 2400 rpm for 10 min and the upper lipid-containing phase was collected. The extraction of the lower phase was repeated by adding 2 mL MTBE/MeOH/H_2_O (10:3:2.5, v/v/v) and centrifuging for 10 min. The two collected lipid-containing phases were combined and evaporated under a gentle N_2_ stream at 40 °C. The residue was dissolved in 1000 µL IPA and stored at − 80 °C. An aliquot of the bacterial lipid extracts was used for LC–MS/MS measurements.

### Phospholipid profiling by LC–MS/MS

LC–MS/MS measurements for phospholipid profiling were conducted using a Q Exactive Plus Orbitrap (Thermo Scientific, Bremen, Germany) hyphenated to an UltiMate 3000 UHPLC system (Thermo Scientific, Dreieich, Germany), which includes a WPS-3000TRS autosampler, a TCC-3000SD column compartment, a DGP-3600RS dual pump, and an SRD-3600 degasser.

Phospholipid profiling by RP-HPLC was performed using an XSelect Premier CSH C_18_ column (100 × 2.1 mm, 2.5 µm; Waters, Milford, MA, USA). Bacterial-lipid separation was achieved using an optimized binary gradient based on A, 10 mM NH_4_OAc (pH 5.5)/MeOH (95:5, v/v) and B, 10 mM NH_4_OAc in MeOH/IPA (60:40, v/v) + 0.01% AA. The binary gradient was initiated at 80%B for 1 min. The organic content was rapidly increased to 92%B within 1 min, followed by a gradual increase to 98%B within 14 min, and finally to 100%B within 0.5 min. The solution was kept at 100%B for 5 min as a rinsing step. The gradient was then adjusted to the starting conditions of 80%B within 0.5 min, which was maintained for 4 min. RP-HPLC was performed for a total runtime of 26 min, with 300 µL/min flow rate, 2 µL injection volume, and 40 °C column compartment temperature.

Lipid ionization on the Q Exactive Plus Orbitrap was performed using a heated electrospray ionization source (HESI-II) in negative ionization mode. For ionization, the following parameters were used: 4 kV spray voltage, 50 arbitrary units (a.u.) sheath gas flow, 15 a.u. auxiliary gas flow, 1 a.u. sweep gas flow, 350 °C heater temperature, 325 °C capillary temperature, and 95 s-lens rf level. Additionally, full MS settings on the Orbitrap were set as follows: *m*/*z* 350–1600 scan range, 70,000 resolution (at *m*/*z* 200), 3e6 AGC target, and 100 ms maximum injection time. For fragmentation, data-dependent acquisition (dda)-MS/MS was performed as a top 5 experiment by higher-energy collision dissociation (HCD) based on the following parameters: 25 eV normalized collision energy (at *m*/*z* 500), ± 1 *m*/*z* isolation width, 10 s dynamic exclusion, 17,500 resolution (at *m*/*z* 200), 1e5 AGC target (minimum 1e3), and 50 ms maximum injection time.

### HPLC fractionation of isomeric lipids

The lipid fractionation was carried out on a Vanquish Flex UHPLC system (Thermo Scientific, Dreieich, Germany), which includes a VF-A40-A dual split sampler FT, a VH-C10-A column compartment H, a VF-P32-A dual pump F, as well as an integrated seven-port valve.

To fractionate an isomerically pure PE species, a complementary approach using HILIC and RP-HPLC was employed. A short HILIC gradient was used to separate PE from other lipid classes (collected *t*_R_ range 5.85–6.45 min). The resulting PE fraction was further purified from isomeric interferences using a PE-focused RP-HPLC gradient elution (collected *t*_R_ range 10.39–10.52 min). The isomerically pure PE species was then prepared for the GC determination of fatty acids as methyl esters.

HILIC separation was performed with slight modification to our previous study [40] using an iHILIC Fusion(+) column (20 × 2.1 mm, 5 µm; HILICON AB, Umeå, Sweden) with a binary gradient based on A, NH_4_OAc (35 mM, pH 5.5, 5% ACN) and B, ACN. The binary gradient was initiated at 97%B for 0.2 min. The organic content was gradually reduced to 75%B within 8 min, followed by a rapid reduction to 60%B within 0.3 min. The eluent was kept at 60%B for 3 min and was then adjusted to the starting conditions of 97%B within 0.5 min, which was maintained for 6 min. This resulted in a total runtime of 18 min, applying 300 µL/min flow rate. Injection volume was set to 10 µL, and column compartment temperature to 40 °C. Fractionation was repeated 15 times until 150 µL bacterial PE was collected.

A PE-focused RP separation was achieved using a binary gradient based on A, 10 mM NH_4_OAc (pH 5.5)/MeOH (95:5, v/v) and B, 10 mM NH_4_OAc in MeOH/IPA (90:10, v/v) + 0.01% AA. By changing the solvent B, the elution strength was significantly reduced, resulting in higher resolution of isomeric PE species. The 26-min gradient elution from the phospholipid profiling was kept, while the injection volume was increased to 5 µL. Fractionation was repeated 18 times until 90 µL bacterial PE species was collected.

### Transesterification for FAME analysis

Samples obtained from the HPLC fractionation were dissolved in 100 µL *n*-hexane and transferred to a reaction tube. Next, the solvent was removed under a gentle N_2_ stream. Transesterification was performed by adding 1 mL of acidic MeOH (1% H_2_SO_4_) and heated to 80 °C for 40 min [9]. After cooling on ice, 2 mL *n*-hexane, 0.5 mL H_2_O, and 0.5 mL saturated NaCl solution were added and shaken vigorously. Then, after phase separation, 1 mL of the resulting FAME solution (organic phase) was reduced to a volume of 100 µL under a gentle N_2_ stream and subjected to GC/MS analysis.

### GC/MS analysis of the lipid fraction

GC/MS analysis was conducted on an 8860/5977B GC/MS system equipped with a 7693A autoinjector (Agilent, Waldbronn, Germany). Helium (purity 99.9990%, Westfalen Company, Münster, Germany) was used as the carrier gas at a constant flow rate of 1 mL/min.

Analyses were performed with an HP-5MS UI capillary column (30 m length, 0.25 mm i.d., 0.25 µm film thickness; Agilent, Waldbronn, Germany). Injections (1 µL) were conducted in splitless mode. The GC oven program started for 1 min at 60 °C. Then, the temperature was increased at 130 °C/min to 180 °C, at 3 °C/min to 250 °C, and finally at 20 °C/min to 300 °C (hold time 5 min). In selected ion monitoring (SIM) mode, *m*/*z* 74, *m*/*z* 79, *m*/*z* 81, *m*/*z* 87, *m*/*z* 88, *m*/*z* 101, *m*/*z* 143, *m*/*z* 213, *m*/*z* 242, *m*/*z* 255, *m*/*z* 256, *m*/*z* 257, and *m*/*z* 270 were recorded from 7 min (end of solvent delay) to the end of the run (41.06 min) [41]. The FAMEs of the lipid fraction were determined in full scan mode (*m*/*z* 50–650) and verified using a standard mix of *iso*-/*anteiso-* and *normal* (straight chain) 15:0-methyl ester (15:0-ME) isomers followed by measurements in GC/MS-SIM mode.

### Data processing

LC–MS/MS data was initially evaluated using Xcalibur 4.2. However, to obtain a detailed comparison of the phospholipid profiles, LC–MS/MS data was reprocessed using the open access MZmine 3.1 software [42]. MS data was processed using the exact mass algorithm. A 10 ppm mass accuracy and a 1e4 noise level were applied for MS1 data extraction, while 10 ppm mass accuracy and 3e3 noise level were used for MS2 data. Mass traces with at least four scans, 10 ppm scan to scan accuracy, 5e4 minimum highest intensity, and 1e4 group intensity threshold were converted into chromatograms using the ADAP chromatogram builder [43]. Chromatograms were smoothed by a factor of 5 using the Savitzky-Golay algorithm. Interfering chromatograms were separated into individual features using the local minimum feature resolver with 90% chromatographic threshold, 0.02 minimum search range, 5e4 minimum height, and 1.3 minimum ratio of peak top to edge for at least four data points. In addition, MS/MS scans were paired based on 0.15 min retention time tolerance and 10 ppm precursor tolerance. Isotopic signals without MS/MS spectrum were removed with 3 ppm mass accuracy and 0.05 min retention time tolerance using the ^13^C isotope filter. Additionally, further isotopes of H, C, N, O, and S were removed using the isotopic peak finder with 3 ppm mass accuracy. Features from different measurements were aligned using the join aligner based on 5 ppm mass accuracy (weight 3) and 0.05 min retention time tolerance (weight 1). Rows without MS/MS spectra and < 6 features were removed using the (multithreaded) peak finder as well as duplicate peaks using the duplicate peak filter in the new average filter mode with 1.5 ppm mass accuracy and 0.035 min retention time tolerance. Lipid annotation was performed using 5 ppm mass accuracy for lyso-PE (LPE), phosphatidylcholines (PC), dimethyl-PE (DMPE), monomethyl-PE (MMPE), PE, PG with 60 MS/MS score, and for CL, lyso-PC (LPC) and phosphatidylinositols (PI) with 30 MS/MS score. The MS/MS score is a relative measure of the match between specific lipid fragments in theory and the actual obtained MS/MS spectrum. MMPE and DMPE were added as custom lipid classes in MZmine 3 (MMPE, lipid backbone formula C_6_H_16_NO_6_P, two acyl chains, [M-H]^−^ headgroup fragment C_4_H_11_NO_4_P (*m*/*z* 168.0420); DMPE, lipid backbone formula C_7_H_18_NO_6_P, two acyl chains, [M-H]^−^ headgroup fragment C_3_H_9_NO_4_P (*m*/*z* 154.0264)).

### Lipid nomenclature

The shorthand notation of Liebisch et al. was used for lipid nomenclature [44]. The total fatty acid composition of a phospholipid (PL) is described by the total number of carbon atoms (x) and double bonds (y), for instance PE 31:0 (PL x:y). Individual fatty acid moieties, highlighted by fragmentation experiments, are specified using an underscore for unknown positions (PE 15:0_16:0; PL x1:y1_x2:y2) or a slash for known positions (PE 15:0/16:0; PL x1:y1/ x2:y2). Note that in conventional CID experiments, the stereospecific location of individual fatty acids is not determined. Therefore, a slash is only used in this work for lipids containing two identical fatty acids, such as PE 15:0/15:0. Furthermore, conventional CID experiments cannot distinguish between lipids containing NCFA and BCFA. Therefore, no distinction was made between NCFA and BCFA on individual fatty acid level. Rather, the total number of bound BCFA was described separately from the shorthand notation.

## Results and discussion

### Bacterial phospholipid analysis

Previous studies on bacterial membrane composition have primarily focused on hydrolyzed fatty acids. For instance, Wiedmaier-Czerny et al. [9] conducted a detailed examination of the fatty acid pattern of six major plant-pathogenic bacteria. However, information on the level of intact phospholipids may offer further insight into the biological background of bacterial membrane formation and stability. In order to improve the understanding of the biological diversity of bacteria, we have performed phospholipid profiling on these six plant-pathogenic bacteria with agricultural relevance.

**Fig. 1:**
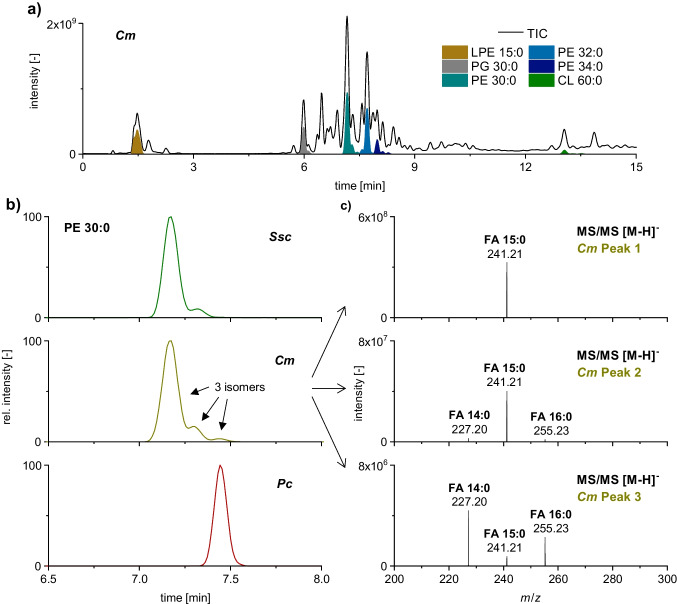
**a** RP–HPLC–MS/MS chromatogram (TIC) of the bacterial lipids in *Cm* including some highlighted lipid species (EIC) from diverse lipid classes. **b** EIC of PE 30:0 lipid species in the bacterial strains *Ssc*, *Cm*, and *Pc* as well as **c** respective MS/MS spectra of the separated lipid species in *Cm*

Using RP-HPLC, the lipid classes in these bacteria were separated by their hydrophobicity, resulting in lipid class–dependent elution windows (Fig. 1a). The main lipid classes observed were PE, PG, CL, and LPE. Monoacylglycerophospholipids such as LPE elute first, followed by the diacylglycerophospholipids PG and PE, and finally four fatty acyl-containing CL. Note that PG and PE species with identical fatty acid combinations exhibit a shift in retention time due to their polar headgroup. Using the XSelect Premier CSH C_18_ column with a bioinert surface, symmetric peak shapes for all lipid classes were achieved. Furthermore, isomeric phospholipid species were detected, including PE 30:0. For this lipid species, three isomers could be chromatographically resolved in *Cm* (Fig. 1b). A comparison with other plant-pathogenic bacteria such as *Ssc* and *Pc* revealed significant differences in the pattern of these PE 30:0 isomers. *Ssc* and *Cm* (both Gram-positive) mainly produced the first eluting isomer, while *Pc* (Gram-negative) exclusively produced the third isomer. Fragmentation experiments of these isomers revealed that the first peak consists of PE 15:0/15:0, the second peak mainly of PE 15:0/15:0 in combination with a smaller amount of the constitutional isomer PE 14:0_16:0 and the third peak mainly of PE 14:0_16:0 in combination with a smaller amount of PE 15:0/15:0 (Fig. 1c). These structural investigations showed the presence of different lipid isomers with identical fatty acid patterns, which can only be distinguished chromatographically and require further investigation for a comprehensive characterization. In addition, a coelution of constitutional isomers was indicated by the fragmentation experiments. However, reference standards are necessary to confirm this assumption.

Wiedmaier-Czerny et al. showed that the fatty acid pattern of *Pc* (abbreviated as *Ac* in the reference) mostly consisted of NCFA [9]. Therefore, it was likely that the third isomer contained two NCFA. Since various studies on BCFA or BCFA-containing lipids have also determined a reduced retention of BCFA in RP-HPLC compared to NCFA [19, 28], we suppose that the detected PE 30:0 isomers differ in the number of bound BCFA. By summing up the probabilities of different fatty acid combinations based on the previous GC study, it may be possible to draw further conclusions about the retention order of these isomeric phospholipids. Therefore, we added the probabilities of NCFA and BCFA combinations for PE 30:0 with a different number of bound BCFA and compared these findings with the relative proportions of the PE 30:0 isomers in our LC study. The isomer distribution of PE 30:0 in *Cm* theoretically results in two BCFA for 92.3% (LC, 86.7%), one BCFA for 4.6% (LC, 10.8%), and no BCFA for 3.1% (LC, 2.5%). For *Ssc*, the shares were as follows: 93.2% (LC, 92.6%), 6.5% (LC, 7.4%), and 0.3% (LC, 0.0%), respectively. This comparison shows remarkable similarities in the isomeric distribution based on the number of BCFA present in PE 30:0. However, further validation with authentic reference standards is required to confirm the retention order suggestion.

### Retention order validation of BCFA containing lipids

**Fig. 2:**
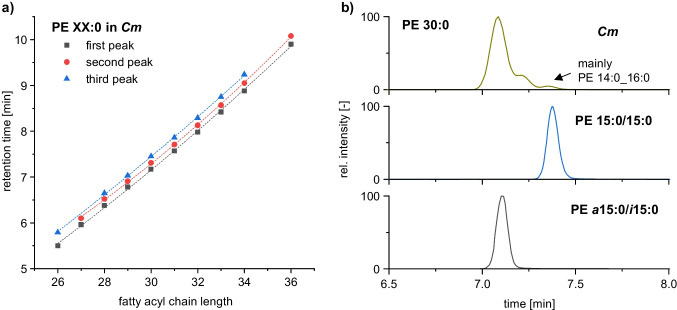
**a** Fatty acyl chain length vs. retention time plot for saturated PE species in *Cm* as well as **b** assignment of methyl-branched lipids by reference standards using RP–HPLC–MS/MS. Therefore, EICs of PE 30:0 in *Cm* (top) of the linear reference standard PE 15:0/15:0 (middle) and of the doubly branched reference standard PE *a*15:0/*i*15:0 (bottom) are plotted

A major limitation in the method development for BCFA containing lipids is the lack of commercially available reference standards. To address this issue, Freeman et al. [19] employed a stable isotope labeling strategy of the amino acid precursors to analyze BCFA containing PG. This study revealed up to three isomers for saturated PG based on the different number of bound BCFA. Thus, a distinction based on bound *a*FA and *i*FA was not achieved. Considering the branching type and the fatty acyl position, up to nine potential isomeric PG species may occur in the sample. Using our developed RP-HPLC method, a separation of up to three PE isomers was achieved as well. As shown in the fatty acyl chain length against retention time plot in Fig. 2a, a polynomial trend in the retention time with increasing chain length is obtained for each isomeric PE species with a coefficient of determination *R*^2^ > 0.999. This systematic behavior indicates the universality of the chromatography for the differentiation of isomeric lipids over a large range of fatty acyl chain length and simplifies the confirmation of isomers after elucidation of the retention order.

To validate the retention order, a complementary approach to Freeman et al. [19] based on reference standards and without the need to use stable isotope labelling was applied in this study. The developed RP-HPLC approach revealed that the reference standard PE 15:0/15:0 and the third PE 30:0 coelute (Fig. 2b). Fragmentation experiments confirmed mainly PE 14:0_16:0 for the third peak in *Cm*, indicating a coelution of constitutional isomers. Recently, the reference standard PE *a*15:0/*i*15:0 has become commercially available and could be used for further validation of the retention order. A coelution of the PE *a*15:0/*i*15:0 standard and the first PE 30:0 isomer was observed. Due to the retention order of the reference standards, it is likely that a separation according to the number of bound BCFA was achieved similar to Freeman et al. [19]. However, the composition of the second isomer had to be verified to confirm this hypothesis. Additionally, it was necessary to determine if lipids containing *i*FA and *a*FA were coeluting. To investigate this point, the second isomer detected in the bacterial strain *Cm* was isolated by HPLC fractionation (see Experimental) and the bound fatty acids were analyzed using GC/MS after transesterification into FAME.

For purification of the second isomer, a complementary HILIC and RP-HPLC fractionation approach was utilized (please refer to Electronic Supplementary Material (ESM) section SI-1). Using HILIC, an efficient separation of lipids is achieved according to their polar head group. Therefore, HILIC fractionation was utilized to purify the PE lipid class from other diacylglycerophospholipids such as PG. This step is particularly crucial, since the hydrolyzed fatty acids of coeluting PG may interfere with those of PE in the GC/MS analysis. To obtain an isomerically pure PE species, the PE fraction was further fractionated by RP-HPLC. For this purpose, the chromatographic resolution of PE in the RP-HPLC method was modified by reduction of the elution strength, resulting in the elution of CL in the rinsing step. Instead of PE 30:0, the second isomer of PE 29:0 was fractionated as it had the highest abundance in *Cm*, leading to a higher isomeric purity after fractionation. In addition, phospholipids with odd-numbered chains, such as PE 29:0 (with 14:0 and 15:0 isomers), have a lower number of constitutional isomers. The varying fatty acids of PE 14:0_15:0 also facilitated a straightforward identification in GC/MS. Futhermore, the fatty acids of the fractionated lipid species PE 14:0_15:0 are not ubiquitous, which improves the informative value of the GC/MS results. After fractionation, an isomer purity of > 99.9% for PE 14:0_15:0 was obtained and negligible interferences from coeluting lipid species that could interfere in the GC/MS analysis (such as PE 14:0_16:1: < 2% and PE 15:0_16:1: < 0.2%) were detected using LC–MS/MS for the fractionated PE 14:0_15:0 isomer.

**Fig. 3:**
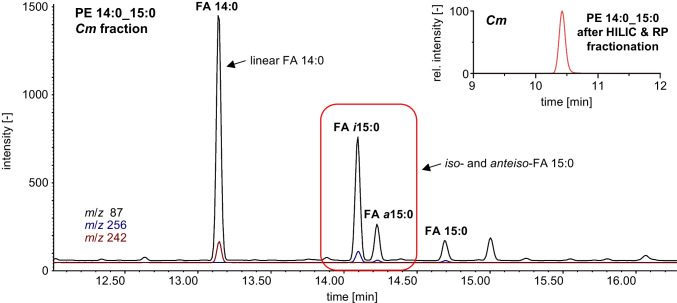
Overlayed GC/MS-SIM chromatograms of FAMEs present in the transmethylated PE 14:0_15:0 (second peak) as fractionated by HPLC from the bacterial strain *Cm* (With: *m*/*z* 87, quantification ion for saturated FAME; *m*/*z* 256, M^+^ of 14:0-ME; *m*/*z* 242, M^+^ of 15:0 ME isomers) [41]

The GC/MS analysis of the fractionated lipid was conducted based on the study of Wiedmaier-Czerny et al. [9]. The availability of various BCFA reference standards in combination with the high chromatographic resolution allowed for a precise characterization of the bound fatty acids of the unknown PE isomer. Using GC/MS, the presence of NCFA 14:0 in the fractionated PE 14:0_15:0 species was revealed, while FA 15:0 was detected as both *i*FA and *a*FA (Fig. 3). This confirmed the coelution of *anteiso*- and *iso*-containing PE. Furthermore, the retention order from Freeman et al. [19] was validated with the additional GC/MS measurement. Thus, based on the retention order, the first isomer contained two BCFA, the second one BCFA, and the third no BCFA, respectively.

### Transfer to other lipid classes

**Fig. 4:**
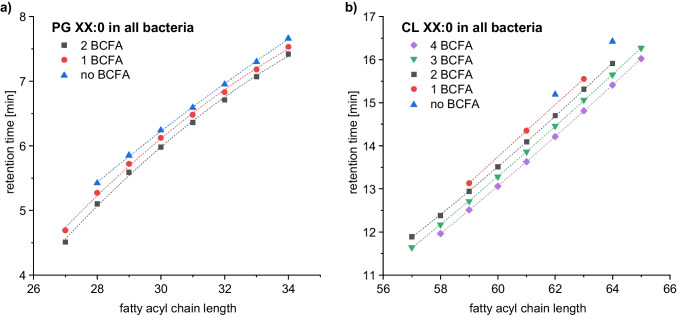
Fatty acyl chain length vs. retention time plot for **a** saturated PG species and **b** saturated CL species detected in all bacteria using RP–HPLC–MS/MS

Since the aim of this study was to perform a comprehensive phospholipid profiling of plant-pathogenic bacteria, the transferability of the results for saturated PE to other lipid classes and unsaturated lipids was explored in the following. To investigate the isomerism of different lipid classes, the identified saturated PG and CL were plotted as a function of the fatty acyl chain length and the retention time (Fig. 4). Analogous to PE, three isomers based on the number of bound BCFA were detected for the diacylglycerophospholipids of PG. However, for CL, up to five isomers could be revealed. Since CL contain four fatty acyls, the detection of five isomers is in accordance with the validated retention order. Thus, the first eluting isomer contained four BCFA, the second three BCFA, the third two BCFA, the fourth one BCFA, and the fifth no BCFA, respectively. As a further confirmation, only CL without BCFA were determined in the bacterial strain *Pc*.

**Fig. 5:**
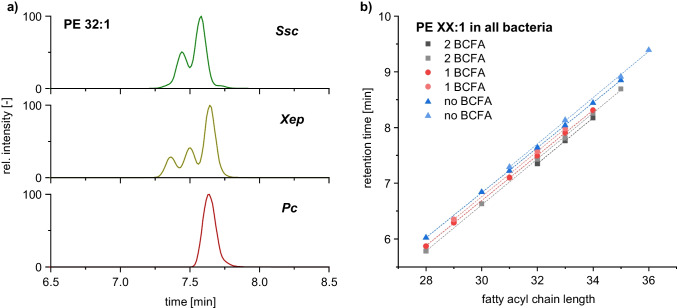
**a** EIC of PE 32:1 lipid species in the bacterial strains *Ssc*, *Xep*, and *Pc* as well as **b** fatty acyl chain length vs. retention time plot for monounsaturated PE species (including different double bond positions) detected in all bacteria using RP–HPLC–MS/MS. Lipids with a systematic shift in retention time were identified (presumably due to differences in double bond position). However, the exact double bond positions could not be validated using conventional LC–MS/MS experiments

For unsaturated lipids, the retention order becomes more complicated. A slight retention time shift was detected between the PE 32:1 species in the bacterial strains *Ssc* and *Xep* (Fig. 5a). The double bond position of the unsaturated fatty acid may be responsible for this. Wiedmaier-Czerny et al. [9] found mainly ∆7 double bond positions in *Xep* and *Pc*. The PE 32:1 species without BCFA also coeluted in both bacterial strains. For *Ssc*, the double bond position was not revealed. However, the PE 32:1 species with one BCFA in *Ssc* coeluted with a species in *Cm*, where the major double bond position was ∆9. Therefore, we suggest a difference in the double bond position of *Ssc* and *Xep*. The fatty acyl chain length against the retention time plot for monounsaturated PE also revealed a systematic behavior (Fig. 5b). Similar retention behavior was also observed for monounsaturated PG and CL (please refer to Electronic Supplementary Material (ESM) section SI-2). However, this assumption could not be validated in the study, as further structural elucidation of the exact double bond position of the lipids is required. Nevertheless, in the subsequent profiling of plant-pathogenic bacteria, unsaturated lipid species with a clear systematic shift in retention time were identified as distinct lipid species.

### Application on six plant-pathogenic bacteria

With the acquired knowledge regarding the retention behavior, the phospholipid profiles of six plant-pathogenic bacteria (*Ssc*, *Xep*, *Xcc*, *Cm*, *Pc*, and *Pst*) were structurally elucidated using LC–MS/MS (ESM section SI-3 and section “Identified bacterial lipids”). Only species with plausible retention time and fragmentation were annotated. In the case of constitutional isomers, the fatty acid combination with the highest abundance was always used for annotation. A total of 297 phospholipid species were identified in the six bacterial strains, with the majority containing BCFA (195 species, 66%). In addition to the main lipid classes CL (127 species), PE (74 species), PG (56 species), and LPE (15 species), also some minor lipid classes were detected, including PC (12 species), MMPE (5 species), LPC (3 species), PI (3 species), and DMPE (2 species). Note that the choline-based lipid classes PC and LPC as well as its biological intermediates MMPE and DMPE were almost exclusively detected in the bacterial strains *Pst* and *Xcc*.

The fatty acid pattern of the identified phospholipids was consistent with the previous study by Wiedmaier-Czerny et al. [9] Specifically, phospholipids carried only MUFA next to NCFA and BCFA. In addition, Wiedmaier-Czerny et al. [9] found differences in the composition of NCFA and BCFA in plant-pathogenic bacteria, highlighting the absence of BCFA in *Pc* and *Pst*. It is therefore of great interest to determine whether these results can be extrapolated to intact phospholipids and if additional insights can be gained from the information about the bound lipid class. For this purpose, differences in the amount of bound BCFA for lipids from varying lipid classes and with varying double bond number were further investigated (ESM section SI-4).

**Fig. 6:**
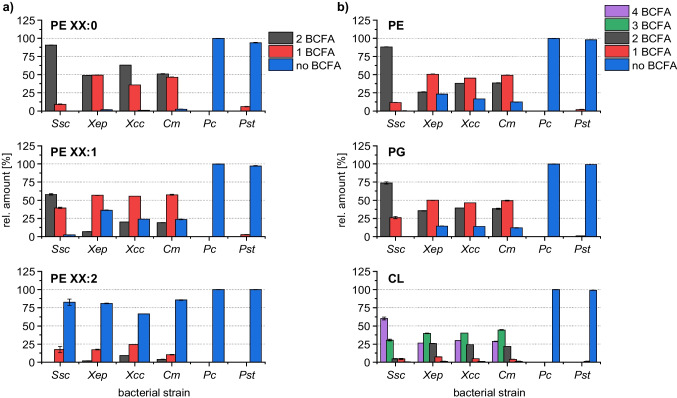
Detailed comparison of the lipid composition of six plant-pathogenic bacteria depending on the degree of bound BCFA for **a** saturated PE, monounsaturated PE, and doubly unsaturated PE as well as **b** the overall composition for the lipid classes PE, PG, and CL using LC–MS/MS

Distinct differences in the PE composition were observed depending on the number of bound BCFA among the plant-pathogenic bacteria (Fig. 6a). In accordance with the findings of Wiedmaier-Czerny et al. [9], *Pc* and *Pst* produce almost exclusively PE without BCFA (*Pc* > 99%, *Pst* > 94% without BCFA). Furthermore, for saturated PE, mainly species with one or two bound BCFA were detected in *Xep*, *Xcc*, and *Cm* (two BCFA, 49–63%; one BCFA, 36–49%; no BCFA, 1–2%), while in *Ssc*, saturated PE species primarily featured two BCFA (two BCFA, 91%; one BCFA, 9%; no BCFA, 0%). As the number of double bonds in the PE species increases, the proportion of bound BCFA decreases. For instance, saturated PE in *Cm* have one or two BCFA bound (two BCFA, 51%; one BCFA, 47%; no BCFA, 2%), while monounsaturated PE have mainly species with one BCFA (two BCFA, 19%; one BCFA, 57%; no BCFA, 24%), and doubly unsaturated PE mostly without BCFA (two BCFA, 4%; one BCFA, 10%; no BCFA, 86%). This behavior was also observed in *Ssc* (saturated two BCFA, 91%; one BCFA, 9%; no BCFA, 0%; monounsaturated two BCFA, 58%; one BCFA, 40%; no BCFA, 2%; doubly unsaturated two BCFA, 0%; one BCFA, 17%; no BCFA, 83%). Here, it is noteworthy that *Cm* and *Ssc* were the only two Gram-positive bacteria analyzed in this study. The reason for this behavior was that the MUFA in the investigated bacteria are typically present as NCFA [9]. Therefore, only a small amount of doubly unsaturated PE were detected with bound BCFA.

Further, the overall composition of PE depending on the degree of bound BCFA was investigated and compared with PG and CL (Fig. 6b). For PG and PE, a similar distribution has been found. Species without BCFA were mainly observed in *Pc* and *Pst* (PE, > 98% without BCFA; PG, > 99% without BCFA), while *Xep*, *Xcc*, and *Cm* showed a distribution over all different number of BCFA (PE, 26–39% two BCFA, 45–51% one BCFA, 12–23% no BCFA; PG, 36–40% two BCFA, 46–50% one BCFA, 12–14% no BCFA). In *Ssc*, mainly species with two bound FA were detected (PE, 88% two BCFA, 12% one BCFA, 0% no BCFA; PG, 74% two BCFA, 26% one BCFA, 0% no BCFA). A similar composition was observed in CL. Again, *Pc* and *Pst* contain almost exclusively species without BCFA (*Pc* > 99%, Pst > 98% without BCFA), while *Ssc* mainly produced species with the highest number of bound BCFA (60% four BCFA, 30% three BCFA, 5% two BCFA, 4% one BCFA, 0% no BCFA). However, *Xep*, *Xcc*, and *Cm* had a distribution that covered CL with two to four bound BCFA (26–30% four BCFA, 40–44% three BCFA, 22–26% two BCFA, 4–7% one BCFA, 1% no BCFA). Thus, only a small amount of CL with less than two bound BCFA were detected in these bacterial strains. In contrast, species without BCFA were detected consistently for the diacylglycerophospholipids PG and PE in *Xep*, *Xcc*, and *Cm*.

The phospholipid profiling revealed significant differences among three groups of bacterial strains: *Pc* ~ *Pst*, *Xep* ~ *Xcc* ~ *Cm*, and *Ssc*. The absence of BCFA in *Pc* and *Pst* by the previous fatty acid study [9] was confirmed for intact phospholipids. Additionally, the preference of *Ssc* to synthesize lipids with multiple BCFA provided further information on the biological background of bacterial membranes as well as the higher preference of CL to bound BCFA compared to PE and PG in *Xep*, *Xcc*, and *Cm*. The results of this work indicate the importance of investigating intact bacterial phospholipids in addition to common fatty acid studies.

## Conclusion

Although various plant-pathogenic bacteria significantly influence food production efficiency, there is a lack of studies on the characterization of cellular lipids. This is due to the complexity of the bacterial lipidome, including isomers based on the double bond position and on bound BCFA. In this study, we examined the phospholipid profile of the plant-pathogenic bacteria *Pc*, *Cm*, *Pst*, *Ssc*, *Xcc*, and *Xep* using an RP–HPLC–MS/MS method with a focus on methyl-branched species.

The developed method achieved a separation based on the number of bound BCFA, resulting in three isomers for the diacylglycerophospholipids PG and PE, and five isomers for four fatty acyl-containing CL. In addition, the number of isomer species in each class of unsaturated phospholipids was twice as high compared to the saturated lipids, presumably due to varying double bond positions. Based on the limited availability of suitable reference standards, the validation of the retention order was challenging. However, the HPLC fractionation of individual isomers followed by GC/MS analysis of the hydrolyzed fatty acids has proven to be a suitable method for retention order validation in the absence of reference standards. A comparison of the six plant-pathogenic bacteria based on the number of bound BCFA revealed similarities, but also interesting differences among three groups of bacterial strains: *Pc* ~ *Pst*, *Xep* ~ *Xcc* ~ *Cm*, and *Ssc*. Especially the preference of *Ssc* to synthesize lipids with multiple BCFA provided additional information to common FA studies.

Examining the phospholipid profile proved to be important in differentiating various bacteria. Our validated retention order simplifies the phospholipid analysis of bacteria producing BCFA in further studies and has potential to aid in automated annotation of isomeric lipids based on universal retention time dependencies. Thus, the extension on BCFA bound phospholipids in lipidomics may provide further insight into the biological background of bacterial membrane formation, stability, and susceptibility.

## Supplementary Information

Below is the link to the electronic supplementary material.

Supplementary file1 (PDF 595 KB)

Supplementary file2 (XLSX 219 KB)

## References

[1] Oerke E-C, Dehne H-W. Safeguarding production—losses in major crops and the role of crop protection. Crop Prot. 2004;23:275–85. 10.1016/j.cropro.2003.10.001.

[2] Loria R, Kers J, Joshi M. Evolution of plant pathogenicity in Streptomyces. Annu Rev Phytopathol. 2006;44:469–87. 10.1146/annurev.phyto.44.032905.091147. 16719719 10.1146/annurev.phyto.44.032905.091147

[3] Lerat S, Simao-Beaunoir A-M, Beaulieu C. Genetic and physiological determinants of Streptomyces scabies pathogenicity. Mol Plant Pathol. 2009;10:579–85. 10.1111/j.1364-3703.2009.00561.x. 19694949 10.1111/j.1364-3703.2009.00561.x PMC6640508

[4] Cuppels DA. Generation and characterization of Tn5 insertion mutations in Pseudomonas syringae pv. tomato. Appl Environ Microb. 1986;51:323–7. 10.1128/aem.51.2.323-327.1986. 10.1128/aem.51.2.323-327.1986 PMC238867 16346988

[5] Xin X-F, He SY. Pseudomonas syringae pv. tomato DC3000: a model pathogen for probing disease susceptibility and hormone signaling in plants. Annu Rev Phytopathol. 2013;51:473–98. 10.1146/annurev-phyto-082712-102321. 23725467 10.1146/annurev-phyto-082712-102321

[6] Hayouka Z, Bella A, Stern T, Ray S, Jiang H, Grovenor CRM, Ryadnov MG. Binary encoding of random peptide sequences for selective and differential antimicrobial mechanisms. Angew Chem Int Edit. 2017;56:8099–103. 10.1002/anie.201702313. 10.1002/anie.201702313 28557193

[7] Amso Z, Hayouka Z. Antimicrobial random peptide cocktails: a new approach to fight pathogenic bacteria. Chem Commun. 2019;55:2007–14. 10.1039/c8cc09961h. 10.1039/c8cc09961h 30688322

[8] Topman S, Tamir-Ariel D, Bochnic-Tamir H, Stern Bauer T, Shafir S, Burdman S, Hayouka Z. Random peptide mixtures as new crop protection agents. Microb Biotechnol. 2018;11:1027–36. 10.1111/1751-7915.13258. 29488347 10.1111/1751-7915.13258 PMC6196386

[9] Wiedmaier-Czerny N, Schroth D, Topman-Rakover S, Brill A, Burdman S, Hayouka Z, Vetter W. Detailed analysis of the fatty acid composition of six plant-pathogenic bacteria. J Chromatogr B. 2021;1162:122454. 10.1016/j.jchromb.2020.122454. 10.1016/j.jchromb.2020.122454 33373896

[10] Mansfield J, Genin S, Magori S, Citovsky V, Sriariyanum M, Ronald P, et al. Top 10 plant pathogenic bacteria in molecular plant pathology. Mol Plant Pathol. 2012;13:614–29. 10.1111/j.1364-3703.2012.00804.x. 22672649 10.1111/j.1364-3703.2012.00804.x PMC6638704

[11] Kaneda T. Iso- and anteiso-fatty acids in bacteria: biosynthesis, function, and taxonomic significance. Microbiol Rev. 1991;55:288–302. 10.1128/mr.55.2.288-302.1991. 1886522 10.1128/mr.55.2.288-302.1991 PMC372815

[12] Rilfors L. Difference in packing properties between iso and anteiso methyl-branched fatty acids as revealed by incorporation into the membrane lipids of Acholeplasma laidlawii strain A. BBA-Biomembranes. 1985;813:151–60. 10.1016/0005-2736(85)90228-7.

[13] Lindström F, Thurnhofer S, Vetter W, Gröbner G. Impact on lipid membrane organization by free branched-chain fatty acids. Phys Chem Chem Phys. 2006;8:4792–7. 10.1039/b607460j. 17043723 10.1039/b607460j

[14] Dowhan W. Molecular basis for membrane phospholipid diversity: why are there so many lipids? Annu Rev Biochem. 1997;66:199–232. 10.1146/annurev.biochem.66.1.199. 9242906 10.1146/annurev.biochem.66.1.199

[15] Fahy E, Subramaniam S, Brown HA, Glass CK, Merrill AH, Murphy RC, et al. A comprehensive classification system for lipids. J Lipid Res. 2005;46:839–61. 10.1194/jlr.E400004-JLR200. 15722563 10.1194/jlr.E400004-JLR200

[16] Liebisch G, Fahy E, Aoki J, Dennis EA, Durand T, Ejsing CS, et al. Update on LIPID MAPS classification, nomenclature, and shorthand notation for MS-derived lipid structures. J Lipid Res. 2020;61:1539–55. 10.1194/jlr.S120001025. 33037133 10.1194/jlr.S120001025 PMC7707175

[17] Rühl J, Hein E-M, Hayen H, Schmid A, Blank LM. The glycerophospholipid inventory of Pseudomonas putida is conserved between strains and enables growth condition-related alterations. Microb Biotechnol. 2012;5:45–58. 10.1111/j.1751-7915.2011.00286.x. 21895997 10.1111/j.1751-7915.2011.00286.x PMC3815271

[18] Fang J, Barcelona MJ. Structural determination and quantitative analysis of bacterial phospholipids using liquid chromatography/electrospray ionization/mass spectrometry. J Microbiol Meth. 1998;33:23–35. 10.1016/s0167-7012(98)00037-2.

[19] Freeman C, Hynds HM, Carpenter JM, Appala K, Bimpeh K, Barbarek S, et al. Revealing fatty acid heterogeneity in staphylococcal lipids with isotope labeling and RPLC-IM-MS. J Am Soc Mass Spectrom. 2021;32:2376–85. 10.1021/jasms.1c00092. 34014662 10.1021/jasms.1c00092 PMC10227724

[20] Kaneda T. Fatty acids in the genus Bacillus. I. Iso- and anteiso-fatty acids as characteristic constituents of lipids in 10 species. J Bacteriol. 1967;93:894–903. 10.1128/jb.93.3.894-903.1967. 4960925 10.1128/jb.93.3.894-903.1967 PMC276533

[21] Gutnikov G. Fatty acid profiles of lipid samples. J Chromatogr B. 1995;671:71–89. 10.1016/0378-4347(95)00116-z. 10.1016/0378-4347(95)00116-z 8520704

[22] Wang DH, Wang Z, Brenna JT. Gas chromatography chemical ionization mass spectrometry and tandem mass spectrometry for identification and straightforward quantification of branched chain fatty acids in foods. J Agr Food Chem. 2020;68:4973–80. 10.1021/acs.jafc.0c01075. 32298092 10.1021/acs.jafc.0c01075

[23] Cajka T, Fiehn O. Comprehensive analysis of lipids in biological systems by liquid chromatography-mass spectrometry. Trend Anal Chem. 2014;61:192–206. 10.1016/j.trac.2014.04.017. 10.1016/j.trac.2014.04.017 PMC4187118 25309011

[24] Lange M, Ni Z, Criscuolo A, Fedorova M. Liquid chromatography techniques in lipidomics research. Chromatographia. 2019;82:77–100. 10.1007/s10337-018-3656-4.

[25] Vosse C, Wienken C, Cadenas C, Hayen H. Separation and identification of phospholipids by hydrophilic interaction liquid chromatography coupled to tandem high resolution mass spectrometry with focus on isomeric phosphatidylglycerol and bis(monoacylglycero)phosphate. J Chromatogr A. 2018;1565:105–13. 10.1016/j.chroma.2018.06.039. 29983166 10.1016/j.chroma.2018.06.039

[26] Peterka O, Maccelli A, Jirásko R, Vaňková Z, Idkowiak J, Hrstka R, et al. HILIC/MS quantitation of low-abundant phospholipids and sphingolipids in human plasma and serum: dysregulation in pancreatic cancer. Anal Chim Acta. 2024;1288:342144. 10.1016/j.aca.2023.342144. 38220279 10.1016/j.aca.2023.342144

[27] Helmer PO, Behrens A, Rudt E, Karst U, Hayen H. Hydroperoxylated vs dihydroxylated lipids: differentiation of isomeric cardiolipin oxidation products by multidimensional separation techniques. Anal Chem. 2020;92:12010–6. 10.1021/acs.analchem.0c02605. 32867498 10.1021/acs.analchem.0c02605

[28] Fu X, Hafza N, Götz F, Lämmerhofer M. Profiling of branched chain and straight chain saturated fatty acids by ultra-high performance liquid chromatography-mass spectrometry. J Chromatogr A. 2023;1703:464111. 10.1016/j.chroma.2023.464111. 37262934 10.1016/j.chroma.2023.464111

[29] Hauff S, Hottinger G, Vetter W. Enantioselective analysis of chiral anteiso fatty acids in the polar and neutral lipids of food. Lipids. 2010;45:357–65. 10.1007/s11745-010-3400-9. 20221853 10.1007/s11745-010-3400-9

[30] Geibel C, Zhang L, Serafimov K, Gross H, Lämmerhofer M. Towards enantioselective ultrahigh performance liquid chromatography-mass spectrometry-based metabolomics of branched-chain fatty acids and anteiso-fatty acids under reversed-phase conditions using sub-2-μm amylose- and cellulose-derived chiral stationary phases. Chirality. 2022;34:484–97. 10.1002/chir.23413. 35032056 10.1002/chir.23413

[31] Geibel C, Olfert M, Knappe C, Serafimov K, Lämmerhofer M. Branched medium-chain fatty acid profiling and enantiomer separation of anteiso-forms of teicoplanin fatty acyl side chain RS3 using UHPLC-MS/MS with polysaccharide columns. J Pharmaceut Biomed. 2023;224:115162. 10.1016/j.chroma.2023.464111. 10.1016/j.jpba.2022.115162 36423498

[32] Mueller P, Bonner R, Hopfgartner G. Controlled formation of protonated and radical cation precursor ions by atmospheric pressure photoionization with μLC-MS enables electron ionization and MS/MS library search. Anal Chem. 2022;94:12103–10. 10.1021/acs.analchem.2c02105. 36001638 10.1021/acs.analchem.2c02105

[33] Pham HT, Ly T, Trevitt AJ, Mitchell TW, Blanksby SJ. Differentiation of complex lipid isomers by radical-directed dissociation mass spectrometry. Anal Chem. 2012;84:7525–32. 10.1021/ac301652a. 22881372 10.1021/ac301652a

[34] Pham HT, Trevitt AJ, Mitchell TW, Blanksby SJ. Rapid differentiation of isomeric lipids by photodissociation mass spectrometry of fatty acid derivatives. Rapid Commun Mass Sp. 2013;27:805–15. 10.1002/rcm.6503. 10.1002/rcm.6503 23495027

[35] Randolph CE, Beveridge CH, Iyer S, Blanksby SJ, McLuckey SA, Chopra G. Identification of monomethyl branched-chain lipids by a combination of liquid chromatography tandem mass spectrometry and charge-switching chemistries. J Am Soc Mass Spectrom. 2022;33:2156–64. 10.1021/jasms.2c00225. 36218280 10.1021/jasms.2c00225 PMC10173259

[36] Zhao X, Xia Y. Characterization of fatty acyl modifications in phosphatidylcholines and lysophosphatidylcholines via radical-directed dissociation. J Am Soc Mass Spectrom. 2021;32:560–8. 10.1021/jasms.0c00407. 33444004 10.1021/jasms.0c00407

[37] Palyzová A, Řezanka T. Separation and identification of diacylglycerols containing branched chain fatty acids by liquid chromatography-mass spectrometry. J Chromatogr A. 2021;1635:461708. 10.1016/j.chroma.2020.461708. 33223151 10.1016/j.chroma.2020.461708

[38] Schreiberová O, Krulikovská T, Sigler K, Cejková A, Rezanka T. RP-HPLC/MS-APCI analysis of branched chain TAG prepared by precursor-directed biosynthesis with Rhodococcus erythropolis. Lipids. 2010;45:743–56. 10.1007/s11745-010-3447-7. 20635225 10.1007/s11745-010-3447-7

[39] Matyash V, Liebisch G, Kurzchalia TV, Shevchenko A, Schwudke D. Lipid extraction by methyl-tert-butyl ether for high-throughput lipidomics. J Lipid Res. 2008;49:1137–46. 10.1194/jlr.D700041-JLR200. 18281723 10.1194/jlr.D700041-JLR200 PMC2311442

[40] Rudt E, Feldhaus M, Margraf CG, Schlehuber S, Schubert A, Heuckeroth S, et al. Comparison of data-dependent acquisition, data-independent acquisition, and parallel reaction monitoring in trapped ion mobility spectrometry-time-of-flight tandem mass spectrometry-based lipidomics. Anal Chem. 2023;95:9488–96. 10.1021/acs.analchem.3c00440. 37307407 10.1021/acs.analchem.3c00440

[41] Thurnhofer S, Vetter W. A gas chromatography/electron ionization-mass spectrometry-selected ion monitoring method for determining the fatty acid pattern in food after formation of fatty acid methyl esters. J Agr Food Chem. 2005;53:8896–903. 10.1021/jf051468u. 16277380 10.1021/jf051468u

[42] Schmid R, Heuckeroth S, Korf A, Smirnov A, Myers O, Dyrlund TS, et al. Integrative analysis of multimodal mass spectrometry data in MZmine 3. Nat Biotechnol. 2023;41:447–9. 10.1038/s41587-023-01690-2. 36859716 10.1038/s41587-023-01690-2 PMC10496610

[43] Myers OD, Sumner SJ, Li S, Barnes S, Du X. One step forward for reducing false positive and false negative compound identifications from mass spectrometry metabolomics data: new algorithms for constructing extracted ion chromatograms and detecting chromatographic peaks. Anal Chem. 2017;89:8696–703. 10.1021/acs.analchem.7b00947. 28752754 10.1021/acs.analchem.7b00947

[44] Liebisch G, Vizcaíno JA, Köfeler H, Trötzmüller M, Griffiths WJ, Schmitz G, et al. Shorthand notation for lipid structures derived from mass spectrometry. J Lipid Res. 2013;54:1523–30. 10.1194/jlr.M033506. 23549332 10.1194/jlr.M033506 PMC3646453

