# Supplementary material for: LC–MS/MS-based phospholipid profiling of plant-pathogenic bacteria with tailored separation of methyl-branched species
Source: Anal Bioanal Chem. 2024 Jul 25;416(25):5513–25. doi: 10.1007/s00216-024-05451-1 (PMC11427607; doi:10.1007/s00216-024-05451-1)
Supplement: Supplementary file 1 — Supplementary file1 (PDF 595 KB) [file 216_2024_5451_MOESM1_ESM.pdf]

Supporting Information

LC-MS/MS-based phospholipid profiling of plant-pathogenic bacteria with focused separation of methyl-branched species

Edward Rudt[a], Christian Faist[a], Vera Schwantes[a], Nele Konrad[a], Nina Wiedmaier-Czerny[b], Katja Lehnert[b], Shiri Topman-Rakover[c,d], Aya Brill[c,d], Saul Burdman[d], Zvi Hayouka[c], Walter Vetter[b], Heiko Hayen[a*]

[a] University of Münster, Institute of Inorganic and Analytical Chemistry, Corrensstraße 48, 48149 Münster, Germany

[b] University of Hohenheim, Institute of Food Chemistry (170b), Garbenstraße 28, D-70593 Stuttgart, Germany

[c] Institute of Biochemistry, Food Science and Nutrition, The Robert H. Smith Faculty of Agricultural, Food & Environment, The Hebrew University of Jerusalem, Rehovot, 76100, Israel

[d] Department of Plant Pathology and Microbiology, The Robert H. Smith Faculty of Agricultural, Food & Environment, The Hebrew University of Jerusalem, Rehovot, 76100, Israel

Corresponding author:
* Heiko Hayen
e-mail: heiko.hayen@uni-muenster.de
telephone: +49 251 83-36576
fax: +49 251 83-39936756

SI-1: Lipid fractionation

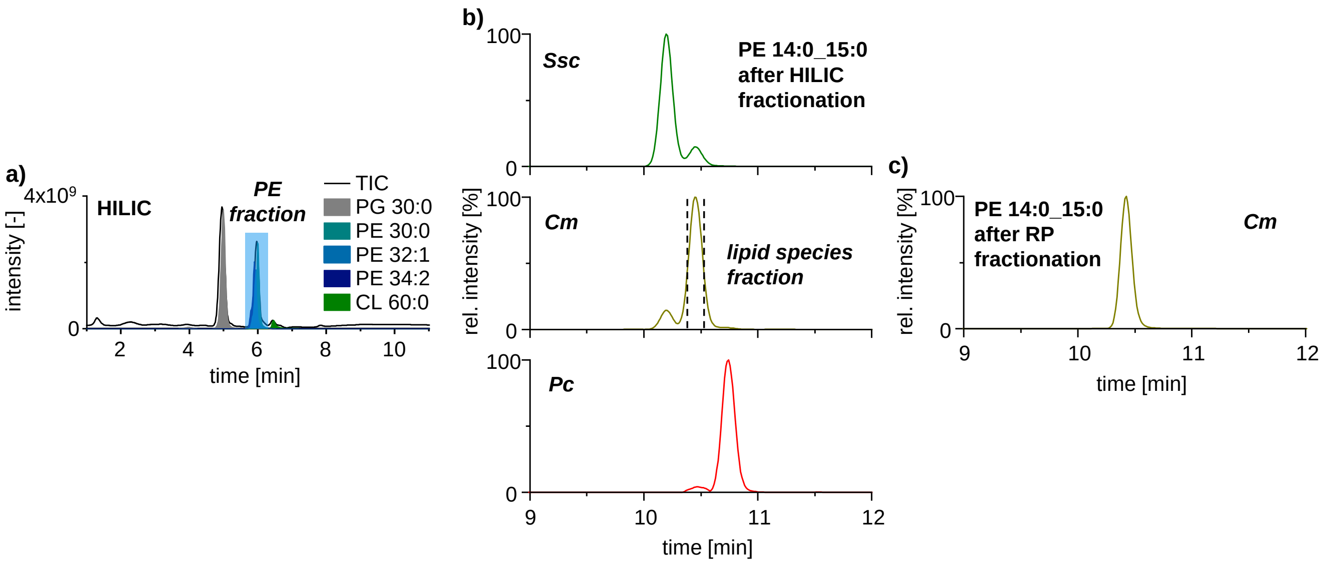

Figure S1: Fractionation of an isomerically pure PE species by a complementary HILIC and RP-HPLC approach. a) HILIC fractionation of the PE lipid class. The TIC of the bacterial lipids including some highlighted lipid species from diverse lipid classes are highlighted (intensities have been adjusted for better visibility). b) RP-HPLC fractionation (10.39 – 10.52 min) of the second PE 14:0_15:0 isomer in *Cm* (PE 14:0_15:0 in *Ssc* and *Pc* are also visualized to track the retention order). The fractionation range was selected to maximize the isomer fraction purity. c) RP-HPLC EIC of the fractionated, isomerically pure PE 14:0_15:0 species.

SI-2: Retention order of monounsaturated PG and CL

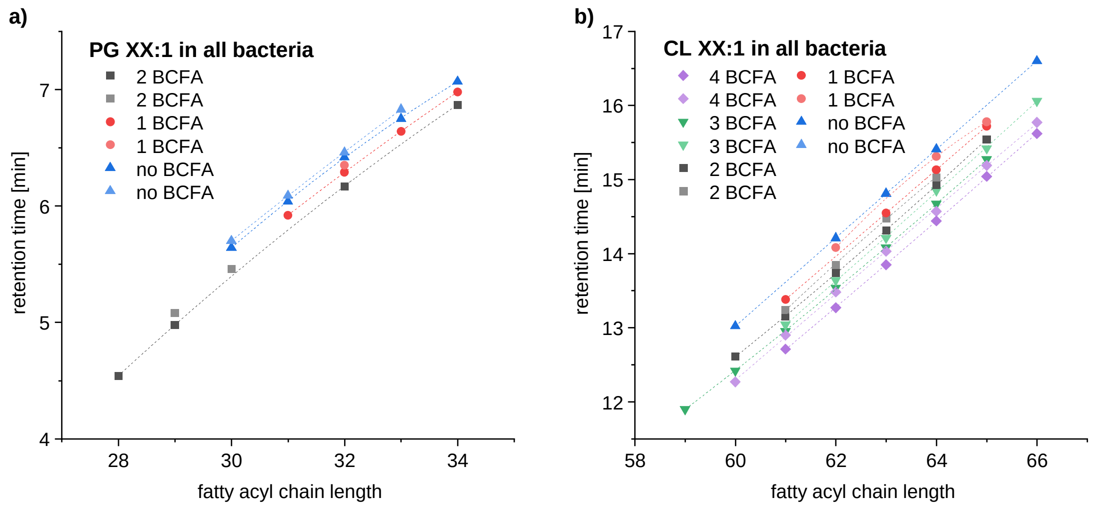

Figure S2: Fatty acyl chain length vs. retention time plot for a) monounsaturated PG species and b) monounsaturated CL species (including different double bond positions) detected in all investigated bacteria using RP-HPLC-MS/MS. Lipids with a systematic shift in retention time were identified (presumably due to differences in double bond position). However, the exact double bond position could not be validated using conventional LC-MS/MS experiments.

SI-3: Identified bacterial phospholipids

Table S1: Identified phospholipids in different plant-pathogenic bacteria, including the number of bound BCFA based on retention time dependencies and the peak area in the individual bacterial strains. Annotation was further based on accurate mass, fragmentation and isotopic distribution. Non-identified lipids are highlighted as “not detected (n.d.)”.

Lipid annotation	*m/z* exp	RT [min]	# BCFA	*Ssc*	*Pc*	*Pst*	*Xep*	*Xcc*	*Cm*	Pool
CL 13:0_14:0/15:0_15:0	1253.8560	11.64	3	n.d.	n.d.	n.d.	2.9E+04	6.8E+03	3.8E+04	9.8E+03
CL 13:0_15:0/13:0_16:0	1253.8561	11.89	2	n.d.	n.d.	n.d.	1.4E+04	n.d.	n.d.	n.d.
CL 14:0_15:0/14:0_15:0	1267.8710	12.38	2	n.d.	n.d.	n.d.	8.6E+04	1.7E+04	1.7E+05	3.6E+04
CL 13:0_15:0/15:0_15:0	1267.8712	12.17	3	n.d.	n.d.	n.d.	6.6E+04	4.0E+04	7.2E+04	2.0E+04
CL 13:0_15:0/15:0_15:0	1267.8713	11.96	4	n.d.	n.d.	n.d.	4.3E+04	3.5E+04	1.5E+05	3.5E+04
CL 14:0_15:0/15:0_15:1	1279.8719	11.90	3	n.d.	n.d.	n.d.	2.7E+04	2.7E+04	1.1E+05	2.3E+04
CL 14:0_15:0/14:0_16:0	1281.8856	12.94	2	n.d.	n.d.	n.d.	1.4E+05	7.2E+04	1.5E+05	3.3E+04
CL 14:0_15:0/15:0_15:0	1281.8863	12.71	3	1.3E+04	n.d.	n.d.	6.1E+05	2.9E+05	1.8E+06	3.4E+05
CL 14:0_15:0/15:0_15:0	1281.8865	12.51	4	4.7E+04	n.d.	n.d.	1.1E+05	9.0E+04	2.0E+05	6.1E+04
CL 14:0_15:0/14:0_16:0	1281.8867	13.13	1	n.d.	n.d.	n.d.	5.1E+03	n.d.	2.9E+04	2.7E+03
CL 15:0_15:0/15:0_15:1	1293.8858	12.27	4	3.5E+03	n.d.	n.d.	6.2E+03	4.5E+04	2.8E+05	5.2E+04
CL 14:0_14:0_16:0_16:1	1293.8860	13.02	0	n.d.	1.7E+04	n.d.	n.d.	n.d.	n.d.	4.7E+03
CL 14:0_15:0/15:0_16:1	1293.8861	12.61	2	n.d.	n.d.	n.d.	3.1E+05	2.8E+05	4.2E+05	9.8E+04
CL 14:0_15:0/15:0_16:1	1293.8864	12.42	3	n.d.	n.d.	n.d.	8.3E+04	4.7E+04	5.9E+04	1.6E+04
CL 15:0/15:0/15:0/15:0	1295.8993	13.06	4	2.6E+05	n.d.	n.d.	3.0E+06	1.9E+06	6.4E+06	1.3E+06
CL 14:0_15:0/15:0_16:0	1295.9023	13.51	2	n.d.	n.d.	n.d.	3.0E+05	1.6E+05	9.2E+05	2.0E+05
CL 15:0/15:0/15:0/15:0	1295.9030	13.28	3	7.2E+04	n.d.	n.d.	8.4E+05	8.3E+05	7.7E+05	2.5E+05
CL 15:0_15:1/15:0_16:1	1305.8810	12.49	1	n.d.	n.d.	n.d.	6.9E+04	4.0E+04	4.8E+04	1.6E+04
CL 15:0_15:1/15:0_16:1	1305.8846	12.34	2	n.d.	n.d.	n.d.	2.1E+04	1.4E+04	n.d.	2.4E+03
CL 15:0_15:1/15:0_16:1	1305.8861	12.19	3	n.d.	n.d.	n.d.	n.d.	2.4E+04	2.1E+04	5.4E+03
CL 14:0_17:1/15:0_15:0	1307.8969	12.71	4	5.5E+04	n.d.	n.d.	n.d.	n.d.	n.d.	1.8E+04
CL 14:0_17:1/15:0_15:0	1307.9012	13.04	3	6.0E+04	n.d.	n.d.	n.d.	n.d.	n.d.	n.d.
CL 15:0_15:0/15:0_16:1	1307.9015	12.95	3	n.d.	n.d.	n.d.	1.5E+06	7.9E+05	1.9E+06	5.1E+05
CL 15:0_15:0/15:0_16:1	1307.9017	12.90	4	6.5E+04	n.d.	n.d.	n.d.	n.d.	n.d.	n.d.
CL 14:0_16:0/15:0_16:1	1307.9019	13.24	2	n.d.	n.d.	n.d.	n.d.	n.d.	n.d.	3.7E+04
CL 15:0_15:0/15:0_16:1	1307.9020	13.15	2	n.d.	n.d.	n.d.	4.6E+05	3.7E+05	n.d.	5.5E+04
CL 14:0_15:0/16:0_16:1	1307.9026	13.38	1	n.d.	n.d.	n.d.	1.5E+05	3.8E+04	6.4E+04	2.1E+04
CL 15:0_15:0/15:0_16:0	1309.9161	13.63	4	6.4E+05	n.d.	n.d.	4.8E+05	3.1E+05	4.1E+05	3.1E+05

CL 15:0_15:0/15:0_16:0	1309.9167	13.86	3	1.9E+05	n.d.	n.d.	2.0E+06	9.4E+05	6.0E+06	1.1E+06
CL 14:0_16:0/15:0_16:0	1309.9178	14.35	1	n.d.	n.d.	n.d.	1.2E+04	4.4E+03	6.5E+04	9.3E+03
CL 15:0_15:0/15:0_16:0	1309.9179	14.09	2	1.2E+04	n.d.	n.d.	3.1E+05	2.0E+05	4.1E+05	1.0E+05
CL 14:0_16:1/16:0_16:1	1319.8968	13.27	0	n.d.	4.9E+05	3.4E+04	6.3E+04	7.2E+04	6.7E+04	1.5E+05
CL 15:0_16:1/15:0_16:1	1319.9023	12.87	2	n.d.	n.d.	n.d.	n.d.	n.d.	7.0E+04	2.4E+04
CL 15:0_16:1/15:0_16:1	1319.9027	12.83	2	3.1E+03	n.d.	n.d.	3.1E+05	1.4E+05	n.d.	4.5E+04
CL 15:0_15:0/15:0_17:1	1321.9148	13.27	4	2.4E+05	n.d.	n.d.	2.3E+05	3.1E+05	5.4E+05	2.0E+05
CL 14:0_17:1/15:0_16:0	1321.9160	13.48	4	2.0E+05	n.d.	n.d.	n.d.	n.d.	1.1E+05	8.7E+04
CL 15:0_15:0/15:0_17:1	1321.9172	13.85	2	2.7E+04	n.d.	n.d.	n.d.	n.d.	5.0E+05	n.d.
CL 14:0_17:1/15:0_16:0	1321.9173	13.54	3	n.d.	n.d.	n.d.	4.1E+05	4.2E+05	n.d.	6.8E+04
CL 14:0_17:1/15:0_16:0	1321.9177	13.64	3	2.8E+05	n.d.	n.d.	n.d.	n.d.	n.d.	n.d.
CL 14:0_17:1/15:0_16:0	1321.9177	13.74	2	n.d.	n.d.	n.d.	1.2E+06	5.2E+05	2.1E+06	6.0E+05
CL 14:0_16:0/16:0_16:1	1321.9178	14.21	0	n.d.	2.8E+05	5.1E+04	n.d.	n.d.	n.d.	9.7E+04
CL 14:0_17:1/15:0_16:0	1321.9179	14.08	1	n.d.	n.d.	n.d.	n.d.	n.d.	1.2E+05	1.3E+04
CL 14:0_17:0/15:0_16:0	1323.9308	14.21	4	9.5E+05	n.d.	n.d.	9.6E+05	4.6E+05	1.3E+06	6.3E+05
CL 14:0_17:0/15:0_16:0	1323.9337	14.46	3	2.7E+05	n.d.	n.d.	4.7E+05	2.5E+05	4.1E+05	2.4E+05
CL 15:0_16:0/15:0_16:0	1323.9340	14.70	2	3.0E+04	n.d.	n.d.	3.4E+05	1.3E+05	1.4E+06	2.8E+05
CL 14:0_16:0/16:0/16:0	1323.9341	15.19	0	n.d.	1.6E+04	n.d.	n.d.	n.d.	1.9E+03	n.d.
CL 15:0_16:1/16:1/16:1	1331.8976	12.72	1	2.9E+03	n.d.	n.d.	4.3E+04	1.1E+04	3.2E+03	5.7E+03
CL 15:0_16:1/15:0_17:1	1333.9114	12.92	4	1.3E+04	n.d.	n.d.	n.d.	n.d.	n.d.	2.7E+03
CL 15:0_16:1/16:0_16:1	1333.9160	13.63	1	1.1E+05	7.3E+03	n.d.	3.5E+05	1.4E+05	1.6E+05	1.3E+05
CL 15:0_16:1/15:0_17:1	1333.9182	13.40	2	1.7E+04	n.d.	n.d.	1.5E+05	1.3E+05	n.d.	1.5E+04
CL 15:0_16:1/15:0_17:1	1333.9186	13.18	3	n.d.	n.d.	n.d.	8.1E+04	7.5E+04	n.d.	4.2E+04
CL 15:0_16:1/15:0_17:1	1333.9188	13.24	3	n.d.	n.d.	n.d.	n.d.	4.2E+04	1.4E+05	2.2E+04
CL 15:0_16:1/15:0_17:1	1333.9188	13.29	3	6.1E+04	n.d.	n.d.	n.d.	n.d.	n.d.	1.6E+04
CL 15:0_16:1/15:0_17:1	1333.9205	13.12	4	2.2E+04	n.d.	n.d.	n.d.	n.d.	n.d.	9.3E+03
CL 15:0_16:0/15:0_17:1	1335.9274	13.85	4	4.5E+05	n.d.	n.d.	6.9E+04	6.9E+04	6.5E+04	1.5E+05
CL 15:0_16:0/15:0_17:1	1335.9308	14.03	4	2.8E+05	n.d.	n.d.	n.d.	n.d.	n.d.	n.d.
CL 15:0_16:0/15:0_17:1	1335.9329	14.48	2	7.5E+04	n.d.	n.d.	n.d.	5.6E+04	8.2E+05	2.4E+05
CL 15:0_15:0/16:1_17:0	1335.9333	14.08	3	n.d.	n.d.	n.d.	8.7E+05	5.0E+05	1.6E+06	3.9E+05
CL 15:0_16:0/15:0_17:1	1335.9333	14.31	2	n.d.	n.d.	n.d.	4.3E+05	2.7E+05	n.d.	6.5E+04
CL 15:0_16:0/15:0_17:1	1335.9334	14.21	3	5.0E+05	n.d.	n.d.	n.d.	n.d.	n.d.	1.1E+05
CL 15:0_16:0/16:0_16:1	1335.9337	14.55	1	n.d.	n.d.	n.d.	2.6E+05	8.2E+04	n.d.	n.d.

CL 15:0_16:0/16:0_16:1	1335.9341	14.81	0	n.d.	1.9E+05	n.d.	5.2E+03	n.d.	n.d.	5.2E+04
CL 15:0_16:0/15:0_17:0	1337.9480	14.81	4	6.2E+05	n.d.	n.d.	1.2E+05	5.3E+04	8.5E+04	2.6E+05
CL 15:0_16:0/15:0_17:0	1337.9494	15.31	2	4.6E+03	n.d.	n.d.	1.0E+05	3.1E+04	1.1E+05	2.6E+04
CL 15:0_16:0/15:0_17:0	1337.9495	15.06	3	2.0E+05	n.d.	n.d.	5.2E+05	1.4E+05	8.7E+05	2.6E+05
CL 15:0_16:0/16:0/16:0	1337.9501	15.55	1	n.d.	n.d.	n.d.	6.4E+03	n.d.	3.7E+04	5.2E+03
CL 16:0_16:1/16:1/16:1	1345.9131	13.48	0	n.d.	3.9E+05	2.5E+05	n.d.	n.d.	n.d.	2.7E+05
CL 15:0_16:1/16:0_17:1	1347.9280	14.21	1	8.4E+04	n.d.	n.d.	2.6E+05	1.5E+05	1.7E+05	6.6E+04
CL 15:0_17:1/15:0_17:1	1347.9281	13.49	4	6.7E+04	n.d.	n.d.	7.5E+03	2.0E+04	2.8E+04	5.9E+04
CL 16:0_16:1/16:0_16:1	1347.9297	14.44	0	2.0E+04	7.5E+06	6.0E+06	1.0E+05	2.6E+04	1.5E+05	3.7E+06
CL 15:0_17:1/15:0_17:1	1347.9309	13.64	4	8.9E+04	n.d.	n.d.	n.d.	n.d.	n.d.	1.2E+04
CL 15:0_17:1/16:0_16:1	1347.9310	14.04	2	6.0E+04	n.d.	n.d.	n.d.	n.d.	n.d.	2.9E+04
CL 15:0_16:1/16:0_17:1	1347.9312	13.96	2	n.d.	n.d.	n.d.	3.1E+05	1.4E+05	3.4E+05	1.1E+05
CL 15:0_17:1/15:0_17:1	1347.9336	13.77	3	n.d.	n.d.	n.d.	2.5E+04	4.7E+04	n.d.	n.d.
CL 15:0_17:1/15:0_17:1	1347.9336	13.87	3	1.5E+05	n.d.	n.d.	n.d.	n.d.	n.d.	n.d.
CL 15:0_17:0/15:0_17:1	1349.9436	14.44	4	n.d.	n.d.	n.d.	1.1E+05	1.0E+05	3.1E+05	8.3E+05
CL 15:0_17:0/15:0_17:1	1349.9464	14.67	3	n.d.	n.d.	n.d.	1.7E+05	1.4E+05	5.6E+04	5.6E+04
CL 15:0_16:0/16:0_17:1	1349.9469	14.85	3	4.6E+05	n.d.	n.d.	n.d.	n.d.	1.3E+06	3.2E+05
CL 15:0_16:0/16:1_17:0	1349.9469	14.93	2	n.d.	n.d.	n.d.	4.1E+05	1.6E+05	n.d.	n.d.
CL 15:0_16:0/16:0_17:1	1349.9472	15.03	2	7.0E+04	n.d.	n.d.	n.d.	n.d.	n.d.	4.0E+04
CL 15:0_16:0/16:1_17:0	1349.9477	15.13	1	n.d.	n.d.	n.d.	7.7E+04	3.1E+04	n.d.	n.d.
CL 15:0_17:0/15:0_17:1	1349.9490	14.57	4	3.1E+05	n.d.	n.d.	n.d.	n.d.	n.d.	n.d.
CL 15:0_16:0/16:0_17:1	1349.9499	15.31	1	n.d.	n.d.	n.d.	n.d.	n.d.	1.6E+05	n.d.
CL 16:0/16:0/16:0_16:1	1349.9500	15.41	0	n.d.	1.2E+06	2.9E+05	3.1E+03	n.d.	n.d.	4.3E+05
CL 15:0_17:0/15:0_17:0	1351.9619	15.41	4	3.4E+05	n.d.	n.d.	1.2E+05	3.8E+04	2.1E+05	3.0E+05
CL 15:0_17:0/15:0_17:0	1351.9631	15.65	3	3.5E+04	n.d.	n.d.	7.1E+04	1.6E+04	5.5E+04	1.8E+04
CL 16:0/16:0/16:0/16:0	1351.9654	16.42	0	n.d.	4.3E+04	4.1E+03	n.d.	n.d.	n.d.	7.4E+03
CL 15:0_16:0/16:0_17:0	1351.9658	15.91	2	n.d.	n.d.	n.d.	6.6E+04	6.9E+03	1.2E+05	2.5E+04
CL 16:0_17:0_16:1/16:1	1361.9455	15.04	0	1.5E+04	9.3E+04	3.0E+04	3.8E+04	2.7E+04	2.0E+05	8.7E+04
CL 15:0_16:0_16:1_18:1	1361.9481	14.85	1	4.9E+04	n.d.	n.d.	n.d.	1.4E+04	5.1E+04	n.d.
CL 16:0_16:1/16:1_17:0	1361.9483	14.80	1	1.5E+05	n.d.	1.5E+05	1.3E+05	5.8E+04	8.2E+04	2.1E+05
CL 15:0_17:0_16:1_17:1	1361.9489	14.56	2	n.d.	n.d.	n.d.	6.9E+04	7.5E+04	n.d.	1.9E+04
CL 15:0_16:0_16:1_18:1	1361.9490	14.72	2	n.d.	n.d.	n.d.	n.d.	n.d.	2.9E+05	n.d.
CL 15:0_16:0_17:1_17:1	1361.9492	14.63	2	8.0E+04	n.d.	n.d.	5.0E+04	n.d.	n.d.	2.7E+04

CL 15:0_16:1_17:0_17:1	1361.9497	14.29	4	6.4E+04	n.d.	n.d.	2.4E+04	4.9E+04	1.1E+05	2.5E+04
CL 15:0_16:1_17:0_17:1	1361.9497	14.36	3	n.d.	n.d.	n.d.	4.1E+04	n.d.	4.0E+04	2.3E+04
CL 15:0_15:0_17:1_18:1	1361.9502	14.43	3	2.0E+05	n.d.	n.d.	2.3E+04	1.3E+04	3.0E+04	9.4E+04
CL 15:0_16:0_17:1_17:1	1361.9503	14.23	4	1.8E+05	n.d.	n.d.	2.7E+03	n.d.	n.d.	1.7E+04
CL 15:0_17:0/16:0_17:1	1363.9600	15.04	4	2.6E+05	n.d.	n.d.	1.7E+04	1.3E+04	2.4E+04	7.4E+04
CL 15:0_17:0/16:0_17:1	1363.9629	15.19	4	2.4E+05	n.d.	n.d.	n.d.	n.d.	n.d.	5.4E+04
CL 15:0_17:0/16:1_17:0	1363.9647	15.27	3	n.d.	n.d.	n.d.	1.6E+05	7.2E+04	3.8E+05	1.1E+05
CL 15:0_17:0/16:0_17:1	1363.9649	15.42	3	2.0E+05	n.d.	n.d.	n.d.	n.d.	5.0E+04	4.1E+04
CL 16:0_16:1/16:0_17:0	1363.9653	15.78	1	n.d.	n.d.	2.7E+04	5.3E+04	n.d.	n.d.	n.d.
CL 15:0_16:0/16:0_18:1	1363.9654	15.72	1	n.d.	n.d.	n.d.	n.d.	9.4E+03	3.0E+05	7.5E+04
CL 15:0_17:0/16:0_17:1	1363.9657	15.54	2	n.d.	n.d.	n.d.	8.2E+04	1.7E+04	n.d.	n.d.
CL 15:0_17:0/16:0_17:0	1365.9786	16.02	4	2.0E+04	n.d.	n.d.	n.d.	n.d.	6.4E+03	1.1E+04
CL 15:0_16:0/17:0/17:0	1365.9816	16.27	3	n.d.	n.d.	n.d.	3.9E+04	3.9E+03	8.9E+04	1.7E+04
CL 16:1/16:1/16:1_18:1	1371.9305	13.63	0	n.d.	2.1E+04	6.9E+04	n.d.	n.d.	n.d.	6.6E+04
CL 16:0_16:1/16:1_18:1	1373.9465	14.58	0	n.d.	2.5E+06	5.5E+06	n.d.	n.d.	n.d.	2.4E+06
CL 16:0_17:1/16:0_17:1	1375.9615	14.65	4	7.5E+04	n.d.	n.d.	n.d.	n.d.	2.0E+04	n.d.
CL 16:0_17:1/16:1_17:0	1375.9617	15.42	1	4.2E+04	n.d.	n.d.	5.5E+04	2.2E+04	1.4E+05	n.d.
CL 15:0_18:1/16:0_17:1	1375.9632	14.82	4	1.4E+05	n.d.	n.d.	n.d.	n.d.	n.d.	1.4E+04
CL 15:0_18:1/16:0_17:1	1375.9639	15.04	3	1.4E+05	n.d.	n.d.	n.d.	n.d.	n.d.	2.2E+04
CL 16:0_17:1/16:1_17:0	1375.9639	15.18	2	5.3E+04	n.d.	n.d.	6.7E+04	3.4E+04	n.d.	1.7E+04
CL 15:0_17:1/16:0_18:1	1375.9641	15.11	3	n.d.	n.d.	n.d.	n.d.	n.d.	1.5E+05	5.2E+04
CL 16:0_16:1/16:0_18:1	1375.9642	15.56	0	n.d.	3.7E+05	2.1E+06	n.d.	n.d.	n.d.	8.1E+05
CL 15:0_17:0/17:0_17:1	1377.9768	15.62	4	8.7E+04	n.d.	n.d.	7.7E+03	7.2E+03	6.2E+04	n.d.
CL 15:0_17:0/16:0_18:1	1377.9785	15.77	4	8.9E+04	n.d.	n.d.	n.d.	n.d.	n.d.	1.9E+04
CL 15:0_16:0/17:0_18:1	1377.9810	16.06	3	7.5E+04	n.d.	n.d.	n.d.	n.d.	1.7E+05	4.5E+04
CL 16:0_16:1/16:0_18:0	1377.9813	16.60	0	n.d.	5.3E+03	1.0E+05	n.d.	n.d.	n.d.	4.1E+04
CL 16:1_17:0/16:1_18:1	1387.9665	14.96	0	n.d.	5.9E+04	1.9E+05	n.d.	n.d.	n.d.	6.9E+04
CL 15:0_18:1/17:0_17:1	1389.9809	15.39	4	6.8E+04	n.d.	n.d.	n.d.	n.d.	n.d.	4.4E+03
CL 15:0_18:1/17:0_17:1	1389.9814	15.64	3	6.3E+04	n.d.	n.d.	n.d.	n.d.	n.d.	1.1E+04
CL 16:0_17:1/16:0_18:1	1389.9823	15.79	2	2.8E+04	n.d.	n.d.	n.d.	n.d.	n.d.	3.3E+03
CL 16:1_18:1/16:1_18:1	1399.9626	14.72	0	2.1E+03	1.8E+05	1.1E+06	n.d.	n.d.	n.d.	4.4E+05
CL 16:0_18:1/16:1_18:1	1401.9789	15.70	0	3.1E+03	2.1E+04	8.5E+05	n.d.	n.d.	1.0E+04	3.0E+05
CL 16:0_18:1/16:1_18:0	1403.9965	16.71	0	n.d.	n.d.	1.7E+05	n.d.	n.d.	2.3E+04	5.6E+04

DMPE 16:0_16:1	716.5216	7.56	0	n.d.	n.d.	1.3E+05	7.0E+02	n.d.	4.3E+02	2.5E+04
DMPE 16:0_18:1	744.5533	8.36	0	n.d.	n.d.	1.6E+04	n.d.	n.d.	n.d.	1.8E+03
LPC 16:1	552.3307	1.43	0	n.d.	n.d.	9.8E+03	n.d.	n.d.	n.d.	n.d.
LPC 16:0	554.3461	1.84	0	n.d.	n.d.	1.1E+04	n.d.	n.d.	n.d.	n.d.
LPC 18:1	580.3627	1.94	0	n.d.	n.d.	3.2E+03	n.d.	n.d.	n.d.	n.d.
LPE 13:0	410.2312	1.15	0	n.d.	n.d.	n.d.	8.6E+04	4.6E+04	8.2E+04	4.2E+05
LPE 14:1	422.2316	1.16	0	n.d.	1.0E+04	n.d.	n.d.	1.6E+04	2.9E+04	7.5E+03
LPE 14:0	424.2472	1.34	0	2.4E+04	1.0E+04	n.d.	5.4E+04	1.3E+05	1.4E+06	3.7E+05
LPE 15:1	436.2472	1.26	0	n.d.	4.3E+03	n.d.	7.1E+03	2.2E+05	5.9E+05	1.2E+05
LPE 15:0	438.2622	1.48	0	3.3E+05	n.d.	n.d.	7.4E+05	9.1E+06	4.7E+07	6.5E+06
LPE 16:1	450.2623	1.46	0	n.d.	2.0E+06	4.6E+05	4.3E+05	5.2E+06	1.9E+06	9.4E+05
LPE 16:0	452.2781	1.76	0	1.3E+05	1.0E+05	6.8E+05	2.0E+05	6.3E+05	2.0E+06	5.2E+05
LPE 17:1	464.2779	1.73	0	1.3E+04	n.d.	n.d.	n.d.	n.d.	2.3E+06	1.9E+05
LPE 17:1	464.2784	1.66	1	3.4E+03	n.d.	n.d.	1.3E+05	4.5E+06	3.6E+06	5.1E+05
LPE 17:0	466.2938	1.91	1	9.7E+03	n.d.	n.d.	1.1E+04	1.2E+05	5.6E+05	7.3E+04
LPE 17:0	466.2938	2.11	0	9.6E+04	n.d.	8.0E+03	1.5E+05	3.4E+05	5.0E+05	1.4E+05
LPE 18:1	478.2938	1.83	1	n.d.	n.d.	2.6E+04	3.9E+03	9.4E+04	1.6E+06	1.9E+05
LPE 18:1	478.2943	1.99	0	n.d.	3.2E+04	2.9E+05	3.5E+04	1.9E+05	1.1E+06	2.9E+05
LPE 18:0	480.3098	2.70	0	n.d.	n.d.	5.0E+04	n.d.	n.d.	3.5E+04	2.6E+04
LPE 18:0	480.3102	2.46	1	n.d.	n.d.	4.9E+03	n.d.	n.d.	5.0E+04	7.9E+03
MMPE 15:1_16:1	686.4758	6.61	0	n.d.	n.d.	n.d.	n.d.	2.9E+04	n.d.	n.d.
MMPE 16:0_16:1	702.5062	7.64	0	7.8E+02	n.d.	n.d.	8.1E+03	5.3E+05	2.9E+03	4.4E+03
MMPE 17:1/17:1	728.5218	7.66	1	n.d.	n.d.	n.d.	n.d.	4.6E+04	n.d.	n.d.
MMPE 17:1/17:1	728.5219	7.53	2	n.d.	n.d.	n.d.	n.d.	3.7E+04	n.d.	n.d.
MMPE 17:1_18:1	742.5387	8.06	0	n.d.	n.d.	n.d.	n.d.	7.8E+03	n.d.	n.d.
PC 14:0_16:0	764.5425	7.40	0	n.d.	n.d.	6.7E+04	n.d.	n.d.	n.d.	1.7E+04
PC 15:0/15:0	764.5440	7.10	2	n.d.	n.d.	n.d.	n.d.	1.2E+05	n.d.	n.d.
PC 15:0_16:1	776.5449	7.02	1	n.d.	n.d.	n.d.	n.d.	3.4E+04	n.d.	n.d.
PC 15:0_16:0	778.5581	7.64	2	n.d.	n.d.	n.d.	n.d.	6.2E+04	n.d.	n.d.
PC 16:1/16:1	788.5425	6.94	0	n.d.	n.d.	1.4E+05	n.d.	n.d.	n.d.	3.4E+04
PC 16:0_16:1	790.5594	7.57	0	n.d.	n.d.	8.0E+06	n.d.	n.d.	n.d.	1.9E+06
PC 15:0_17:0	792.5732	7.93	2	n.d.	n.d.	n.d.	n.d.	7.3E+04	n.d.	n.d.
PC 16:0/16:0	792.5742	8.25	0	n.d.	n.d.	9.0E+05	n.d.	n.d.	n.d.	1.6E+05
PC 16:1_17:0	804.5749	7.85	0	n.d.	n.d.	1.1E+05	n.d.	1.5E+04	n.d.	2.2E+04
PC 16:1_18:1	816.5755	7.72	0	n.d.	n.d.	2.0E+06	n.d.	n.d.	n.d.	6.0E+05
PC 16:1_18:0	818.5911	8.39	0	n.d.	n.d.	1.7E+06	n.d.	n.d.	n.d.	3.7E+05
PC 16:0_18:0	820.6062	9.20	0	1.9E+03	n.d.	9.1E+04	n.d.	n.d.	n.d.	1.5E+04
PE 10:0_15:0	592.3985	5.12	2	n.d.	n.d.	n.d.	1.9E+03	n.d.	n.d.	n.d.
PE 12:0_14:0	606.4140	5.79	0	n.d.	1.6E+03	n.d.	1.1E+04	n.d.	3.1E+03	3.2E+03
PE 11:0_15:0	606.4141	5.50	2	n.d.	n.d.	n.d.	5.3E+04	1.3E+04	3.0E+04	1.1E+04
PE 12:0_15:0	620.4289	6.10	1	n.d.	n.d.	n.d.	5.3E+05	3.4E+04	2.2E+05	1.0E+05
PE 12:0_15:0	620.4298	5.96	2	n.d.	n.d.	n.d.	1.1E+05	2.7E+03	9.0E+03	3.5E+04
PE 12:0_16:1	632.4290	6.02	0	n.d.	6.2E+04	2.0E+04	7.7E+04	1.3E+04	9.7E+03	4.8E+04
PE 12:0_16:1	632.4292	5.87	1	n.d.	n.d.	n.d.	5.9E+04	1.2E+04	4.0E+03	2.2E+04
PE 13:0_15:1	632.4297	5.78	2	n.d.	n.d.	n.d.	n.d.	5.6E+03	2.4E+04	n.d.

PE 12:0_16:0	634.4447	6.65	0	n.d.	2.0E+05	1.1E+05	4.7E+05	1.1E+04	4.1E+05	1.8E+05
PE 12:0_16:0	634.4450	6.52	1	1.0E+04	n.d.	n.d.	1.6E+06	2.4E+05	3.9E+05	3.4E+05
PE 13:0_15:0	634.4451	6.38	2	4.0E+04	n.d.	n.d.	2.2E+06	4.7E+05	1.9E+06	9.7E+05
PE 14:0_15:1	646.4453	6.35	1	n.d.	n.d.	n.d.	n.d.	1.9E+05	6.8E+05	n.d.
PE 13:0_16:1	646.4454	6.29	1	n.d.	n.d.	n.d.	1.6E+06	1.6E+05	n.d.	4.7E+05
PE 14:0_15:0	648.4603	6.78	2	1.3E+06	n.d.	n.d.	3.2E+06	9.6E+05	2.8E+06	1.7E+06
PE 14:0_15:0	648.4605	7.03	0	n.d.	2.8E+05	n.d.	n.d.	4.9E+04	3.5E+05	n.d.
PE 14:0_15:0	648.4606	6.91	1	1.9E+05	n.d.	1.2E+03	1.9E+07	2.6E+06	2.1E+07	7.0E+06
PE 15:1/15:1	658.4450	6.07	2	n.d.	n.d.	n.d.	n.d.	1.5E+04	5.5E+04	9.5E+03
PE 14:1_16:1	658.4452	6.27	0	n.d.	8.0E+04	1.2E+04	4.8E+04	8.4E+03	3.5E+03	3.2E+04
PE 14:0_16:1	660.4606	6.84	0	9.6E+03	1.0E+07	1.3E+06	5.2E+06	1.1E+06	1.3E+06	3.1E+06
PE 15:0_15:1	660.4610	6.63	2	n.d.	n.d.	n.d.	n.d.	2.3E+06	6.7E+06	1.6E+06
PE 15:0/15:0	662.4748	7.17	2	2.0E+07	n.d.	n.d.	4.0E+07	3.2E+07	7.9E+07	3.8E+07
PE 14:0_16:0	662.4761	7.45	0	n.d.	6.3E+06	2.8E+06	6.7E+05	1.7E+05	2.2E+06	2.1E+06
PE 15:0/15:0	662.4770	7.31	1	1.6E+06	5.0E+03	8.3E+03	1.3E+07	8.4E+06	9.6E+06	4.0E+06
PE 15:1_16:1	672.4603	6.62	0	n.d.	2.7E+04	n.d.	7.4E+05	5.3E+05	3.1E+05	2.1E+05
PE 15:0_16:1	674.4758	7.10	1	n.d.	1.2E+05	7.2E+04	4.3E+07	2.1E+07	1.7E+07	1.4E+07
PE 15:0_16:1	674.4758	7.22	0	n.d.	5.4E+06	1.7E+05	9.5E+06	4.5E+06	n.d.	n.d.
PE 14:0_17:1	674.4761	7.29	0	3.0E+04	n.d.	n.d.	n.d.	n.d.	2.2E+06	n.d.
PE 15:0_16:0	676.4910	7.57	2	4.1E+07	n.d.	n.d.	8.2E+06	3.8E+06	4.7E+06	1.4E+07
PE 15:0_16:0	676.4913	7.71	1	6.7E+06	2.1E+04	3.7E+04	3.2E+07	1.2E+07	5.8E+07	2.0E+07
PE 15:0_16:0	676.4914	7.86	0	n.d.	3.7E+06	2.0E+05	4.4E+05	2.6E+05	n.d.	1.0E+06
PE 16:1/16:1	686.4761	7.01	0	n.d.	1.2E+07	7.6E+06	1.0E+07	3.1E+06	8.2E+05	5.4E+06
PE 15:1_17:1	686.4766	6.81	2	n.d.	n.d.	n.d.	1.1E+04	1.7E+05	1.3E+05	1.4E+04
PE 15:0_17:1	688.4897	7.35	2	n.d.	n.d.	n.d.	8.2E+06	1.1E+07	1.2E+07	3.5E+06
PE 16:0_16:1	688.4912	7.64	0	n.d.	2.6E+08	2.2E+08	3.0E+07	1.0E+07	3.4E+06	1.6E+08
PE 15:0_17:1	688.4913	7.57	1	2.3E+06	n.d.	n.d.	n.d.	n.d.	2.3E+07	n.d.
PE 15:0_17:1	688.4914	7.49	1	n.d.	n.d.	n.d.	1.2E+07	1.1E+07	n.d.	n.d.
PE 15:0_17:1	688.4931	7.43	2	1.0E+06	n.d.	n.d.	n.d.	n.d.	n.d.	n.d.
PE 15:0_17:0	690.5068	7.98	2	3.1E+07	n.d.	n.d.	1.9E+07	7.3E+06	1.7E+07	1.7E+07
PE 15:0_17:0	690.5068	8.13	1	9.8E+05	1.3E+04	1.0E+05	4.3E+06	1.6E+06	2.8E+06	1.2E+06
PE 16:0/16:0	690.5074	8.29	0	n.d.	4.2E+07	1.5E+07	3.9E+05	1.4E+05	1.6E+06	1.3E+07
PE 16:1_17:1	700.4914	7.28	1	n.d.	3.9E+04	2.6E+04	3.6E+06	2.3E+06	1.1E+06	1.1E+06
PE 16:1_17:1	700.4915	7.51	0	3.7E+03	4.6E+04	n.d.	n.d.	3.0E+04	2.1E+06	5.5E+05
PE 16:1_17:1	700.4919	7.37	0	1.8E+04	4.4E+05	2.2E+05	2.2E+06	1.4E+06	n.d.	4.6E+05
PE 16:0_17:1	702.5068	7.76	2	n.d.	n.d.	n.d.	n.d.	1.1E+06	n.d.	n.d.
PE 17:0_16:1	702.5070	7.90	1	n.d.	2.1E+05	8.1E+06	2.7E+07	1.2E+07	2.6E+07	1.2E+07
PE 16:0_17:1	702.5070	7.98	1	1.1E+06	n.d.	n.d.	n.d.	n.d.	n.d.	n.d.
PE 16:0_17:1	702.5072	8.04	0	n.d.	2.5E+06	1.7E+06	7.3E+06	3.3E+06	n.d.	2.0E+06
PE 15:0_18:1	702.5073	7.81	2	2.3E+06	n.d.	n.d.	n.d.	n.d.	n.d.	n.d.
PE 16:0_17:1	702.5081	8.13	0	1.0E+05	n.d.	n.d.	n.d.	n.d.	9.1E+06	2.2E+06
PE 16:0_17:0	704.5231	8.42	2	1.3E+06	n.d.	n.d.	6.7E+05	2.5E+05	5.7E+05	6.4E+05
PE 16:0_17:0	704.5239	8.57	1	1.8E+05	1.7E+04	1.2E+06	3.5E+06	5.3E+05	5.5E+06	1.8E+06
PE 16:0_17:0	704.5242	8.75	0	n.d.	1.8E+05	9.1E+04	1.2E+05	1.8E+04	9.9E+04	1.1E+05
PE 17:1/17:1	714.5058	7.54	2	n.d.	n.d.	n.d.	3.8E+05	7.1E+05	2.3E+05	1.1E+05

PE 16:1_18:1	714.5070	7.77	0	n.d.	9.3E+07	1.2E+08	3.1E+06	9.6E+05	3.1E+06	6.6E+07
PE 17:0_17:1	716.5216	8.17	2	2.9E+05	n.d.	n.d.	2.1E+06	2.2E+06	3.7E+06	1.0E+06
PE 17:0_17:1	716.5229	8.31	1	n.d.	n.d.	n.d.	2.2E+06	1.8E+06	7.0E+05	5.2E+05
PE 16:0_18:1	716.5231	8.44	0	n.d.	7.8E+06	7.3E+07	3.0E+06	7.5E+05	1.1E+07	2.8E+07
PE 16:0_18:1	716.5233	8.26	2	1.0E+06	n.d.	n.d.	n.d.	n.d.	n.d.	n.d.
PE 17:0/17:0	718.5383	8.88	2	2.4E+05	n.d.	3.7E+03	4.8E+05	1.8E+05	2.3E+06	5.1E+05
PE 16:0_18:0	718.5401	9.24	0	n.d.	9.9E+04	2.1E+06	1.7E+04	n.d.	7.7E+04	5.9E+05
PE 17:0/17:0	718.5403	9.05	1	1.3E+04	n.d.	1.4E+04	1.6E+05	3.3E+04	1.7E+05	4.8E+04
PE 16:1_19:1	728.5226	7.91	1	n.d.	n.d.	1.8E+05	n.d.	5.9E+03	n.d.	3.1E+04
PE 17:1_18:1	728.5227	8.16	0	n.d.	n.d.	n.d.	2.9E+05	1.5E+05	1.5E+05	n.d.
PE 17:1_18:1	728.5231	8.06	0	4.2E+04	8.4E+06	2.5E+07	4.3E+05	2.5E+05	1.1E+06	4.3E+06
PE 17:1_18:1	728.5232	8.30	0	n.d.	4.8E+04	9.4E+04	n.d.	7.1E+03	1.2E+06	3.3E+05
PE 17:0_18:1	730.5403	8.69	2	3.1E+05	n.d.	n.d.	n.d.	n.d.	n.d.	n.d.
PE 17:0_18:1	730.5406	8.85	0	6.3E+04	1.2E+04	n.d.	n.d.	n.d.	3.4E+05	n.d.
PE 17:0_18:1	730.5406	8.91	0	n.d.	n.d.	n.d.	1.3E+05	3.9E+04	n.d.	n.d.
PE 18:1/18:1	742.5403	8.60	0	n.d.	1.9E+05	1.7E+06	9.7E+04	n.d.	3.5E+05	5.0E+05
PE 18:1/18:1	742.5406	8.50	1	1.4E+04	n.d.	n.d.	4.9E+03	2.2E+03	n.d.	n.d.
PE 18:0_18:1	744.5553	9.39	0	n.d.	6.9E+03	5.0E+05	1.2E+04	n.d.	n.d.	2.0E+05
PE 17:0_19:0	746.5705	10.08	1	n.d.	n.d.	n.d.	n.d.	n.d.	3.6E+03	n.d.
PE 17:0_19:0	746.5712	9.90	2	n.d.	n.d.	n.d.	n.d.	n.d.	1.1E+05	2.2E+04
PE 16:0_20:0	746.5717	10.30	0	n.d.	n.d.	2.8E+04	n.d.	n.d.	n.d.	6.8E+03
PG 12:0_15:0	651.4252	4.51	2	n.d.	n.d.	n.d.	2.8E+04	n.d.	n.d.	8.7E+03
PG 12:0_15:0	651.4254	4.69	1	n.d.	n.d.	n.d.	2.5E+05	2.5E+03	3.7E+04	3.8E+04
PG 12:0_16:1	663.4256	4.54	2	n.d.	n.d.	n.d.	3.3E+04	n.d.	n.d.	1.9E+03
PG 14:0/14:0	665.4401	5.42	0	n.d.	4.0E+04	7.1E+03	6.8E+04	n.d.	1.3E+04	2.2E+04
PG 13:0_15:0	665.4402	5.10	2	n.d.	n.d.	n.d.	7.8E+05	4.8E+04	1.8E+05	2.9E+05
PG 12:0_16:0	665.4404	5.27	1	n.d.	n.d.	n.d.	7.9E+05	3.3E+04	5.8E+04	9.4E+04
PG 14:0_15:1	677.4402	5.08	2	n.d.	n.d.	n.d.	n.d.	1.9E+04	6.2E+04	1.3E+04
PG 13:0_16:1	677.4404	4.98	2	n.d.	n.d.	n.d.	3.6E+05	5.2E+03	n.d.	8.5E+04
PG 14:0_15:0	679.4545	5.85	0	n.d.	6.4E+04	n.d.	n.d.	n.d.	n.d.	n.d.
PG 14:0_15:0	679.4552	5.72	1	n.d.	n.d.	n.d.	1.2E+07	4.1E+05	4.4E+06	1.9E+06
PG 14:0_15:0	679.4559	5.59	2	n.d.	n.d.	n.d.	1.2E+06	1.1E+05	3.5E+05	3.1E+05
PG 14:1_16:1	689.4400	4.97	0	n.d.	6.8E+03	n.d.	n.d.	n.d.	n.d.	n.d.
PG 14:0_16:1	691.4545	5.70	0	n.d.	n.d.	n.d.	n.d.	n.d.	1.7E+05	n.d.
PG 14:0_16:1	691.4552	5.64	0	n.d.	1.0E+06	9.4E+04	8.9E+05	1.4E+05	1.6E+05	3.5E+05
PG 15:0_15:1	691.4555	5.46	2	n.d.	n.d.	n.d.	n.d.	1.8E+05	4.6E+05	1.6E+05
PG 15:0/15:0	693.4709	5.98	2	1.3E+04	n.d.	n.d.	1.8E+07	7.6E+06	2.9E+07	7.4E+06
PG 14:0_16:0	693.4711	6.24	0	n.d.	1.4E+06	2.3E+05	n.d.	1.6E+04	9.3E+04	2.9E+05
PG 15:0/15:0	693.4716	6.12	1	n.d.	n.d.	n.d.	5.7E+06	1.9E+06	2.7E+06	8.3E+05
PG 15:1_16:1	703.4551	5.42	0	n.d.	n.d.	n.d.	3.2E+04	2.1E+04	2.2E+04	8.4E+03
PG 15:0_16:1	705.4699	6.04	0	n.d.	8.6E+05	n.d.	2.0E+06	8.3E+05	n.d.	n.d.
PG 15:0_16:1	705.4700	6.09	0	n.d.	n.d.	n.d.	n.d.	n.d.	4.6E+05	n.d.
PG 15:0_16:1	705.4706	5.92	1	n.d.	1.9E+04	n.d.	8.4E+06	3.2E+06	3.8E+06	2.0E+06
PG 15:0_16:0	707.4860	6.59	0	n.d.	1.8E+06	2.3E+04	n.d.	2.0E+04	n.d.	2.4E+05
PG 15:0_16:0	707.4864	6.48	1	5.6E+03	n.d.	n.d.	1.7E+07	3.9E+06	3.1E+07	8.8E+06

PG 15:0_16:0	707.4867	6.36	2	2.3E+04	n.d.	n.d.	5.3E+06	8.8E+05	1.7E+06	1.3E+06
PG 16:1/16:1	717.4706	5.93	0	n.d.	n.d.	n.d.	n.d.	n.d.	9.1E+04	n.d.
PG 16:1/16:1	717.4709	5.84	0	n.d.	4.5E+05	7.7E+05	5.9E+05	1.9E+05	n.d.	4.6E+05
PG 15:0_17:1	719.4851	6.17	2	5.3E+03	n.d.	n.d.	1.2E+06	1.4E+06	1.4E+06	3.5E+05
PG 16:0_16:1	719.4856	6.46	0	n.d.	n.d.	n.d.	n.d.	n.d.	1.8E+06	n.d.
PG 16:0_16:1	719.4858	6.42	0	n.d.	6.2E+07	6.6E+07	8.4E+06	2.1E+06	n.d.	3.5E+07
PG 15:0_17:1	719.4868	6.29	1	n.d.	n.d.	n.d.	2.8E+06	1.7E+06	n.d.	n.d.
PG 15:0_17:1	719.4869	6.35	1	6.4E+03	n.d.	n.d.	n.d.	n.d.	5.2E+06	n.d.
PG 15:0_17:0	721.5011	6.71	2	n.d.	n.d.	n.d.	1.5E+07	2.2E+06	9.0E+06	4.4E+06
PG 15:0_17:0	721.5017	6.83	1	n.d.	n.d.	n.d.	2.4E+06	5.0E+05	1.3E+06	3.9E+05
PG 16:0/16:0	721.5018	6.95	0	n.d.	1.8E+07	2.7E+06	2.1E+05	1.4E+04	9.9E+04	3.7E+06
PG 16:1_17:1	731.4856	6.12	1	n.d.	n.d.	n.d.	3.5E+05	1.8E+05	1.3E+05	4.5E+04
PG 16:1_17:1	731.4860	6.18	0	n.d.	3.2E+04	n.d.	2.3E+05	1.2E+05	n.d.	8.0E+04
PG 16:1_17:1	731.4862	6.33	0	n.d.	n.d.	n.d.	n.d.	n.d.	2.9E+05	5.6E+04
PG 16:0_17:1	733.5017	6.83	0	n.d.	n.d.	n.d.	n.d.	n.d.	3.5E+06	8.1E+05
PG 17:0_16:1	733.5018	6.75	0	n.d.	3.6E+05	2.4E+05	2.7E+06	8.1E+05	n.d.	n.d.
PG 17:0_16:1	733.5021	6.64	1	2.8E+03	2.9E+04	9.2E+05	7.5E+06	2.9E+06	7.5E+06	2.4E+06
PG 16:0_17:0	735.5173	7.07	2	n.d.	n.d.	n.d.	4.5E+05	7.5E+04	2.9E+05	1.3E+05
PG 16:0_17:0	735.5177	7.18	1	n.d.	1.5E+04	2.0E+05	1.8E+06	2.2E+05	1.5E+06	6.2E+05
PG 16:0_17:0	735.5184	7.30	0	n.d.	1.1E+05	1.6E+04	4.4E+04	n.d.	n.d.	3.4E+04
PG 16:1_18:1	745.5016	6.53	0	n.d.	8.0E+06	3.7E+07	3.8E+05	9.3E+04	5.8E+05	1.1E+07
PG 17:1/17:1	745.5016	6.71	0	n.d.	n.d.	n.d.	n.d.	n.d.	3.1E+05	7.5E+04
PG 17:1/17:1	745.5020	6.44	1	n.d.	n.d.	n.d.	n.d.	9.3E+04	n.d.	n.d.
PG 17:0_17:1	747.5163	6.87	2	n.d.	n.d.	n.d.	6.3E+05	5.6E+05	1.4E+06	2.8E+05
PG 16:0_18:1	747.5176	7.07	0	n.d.	1.3E+06	2.5E+07	1.7E+06	3.0E+05	5.7E+06	7.5E+06
PG 17:0_17:1	747.5178	6.98	1	n.d.	n.d.	n.d.	1.2E+06	5.3E+05	n.d.	n.d.
PG 17:0/17:0	749.5322	7.42	2	n.d.	n.d.	n.d.	3.4E+05	5.6E+04	9.7E+05	2.1E+05
PG 16:0_18:0	749.5326	7.66	0	n.d.	2.3E+04	7.0E+05	n.d.	n.d.	n.d.	2.1E+05
PG 17:0/17:0	749.5329	7.53	1	n.d.	n.d.	n.d.	9.9E+04	3.6E+03	n.d.	8.7E+03
PG 17:1_18:1	759.5171	6.78	0	n.d.	9.4E+04	5.1E+05	5.5E+04	3.2E+04	2.3E+05	1.1E+05
PG 17:1_18:1	759.5172	6.95	0	n.d.	n.d.	n.d.	8.1E+04	3.0E+03	4.4E+05	1.1E+05
PG 18:1/18:1	773.5329	7.20	0	n.d.	1.5E+04	3.4E+05	n.d.	4.4E+03	1.4E+05	1.6E+05
PI 15:0/15:0	781.4899	5.86	2	4.5E+04	n.d.	n.d.	n.d.	n.d.	n.d.	2.1E+04
PI 15:0_16:0	795.5049	6.24	2	1.9E+05	n.d.	n.d.	n.d.	n.d.	n.d.	9.0E+04
PI 15:0_17:0	809.5188	6.58	2	2.0E+05	n.d.	n.d.	n.d.	n.d.	n.d.	1.4E+05

SI-4: BCFA dependent phospholipid profile

Table S2: Relative comparison of the PE profile in different plant-pathogenic bacteria dependent on the number of bound BCFA for saturated PE (PE XX:0), monounsaturated PE (PE XX:1) and doubly unsaturated PE (PE XX:2).

PE XX:0 profile [%]	*Ssc*	*Pc*	*Pst*	*Xep*	*Xcc*	*Cm*	Pool
2 BCFA	90.8	0.0	0.0	49.1	63.2	51.2	58.2
1 BCFA	9.2	0.1	5.9	49.4	35.9	46.5	28.1
no BCFA	0.0	99.9	94.0	1.4	0.9	2.3	13.7

PE XX:1 profile [%]	*Ssc*	*Pc*	*Pst*	*Xep*	*Xcc*	*Cm*	Pool
2 BCFA	58.1	0.0	0.0	6.8	20.2	19.0	2.7
1 BCFA	39.5	0.1	2.6	56.8	55.9	57.4	11.8
no BCFA	2.4	99.9	97.4	36.4	24.0	23.5	85.5

PE XX:2 profile [%]	*Ssc*	*Pc*	*Pst*	*Xep*	*Xcc*	*Cm*	Pool
2 BCFA	0.0	0.0	0.0	1.8	9.2	3.9	0.2
1 BCFA	17.4	0.0	0.1	17.2	24.2	10.4	1.4
no BCFA	82.6	100.0	99.9	81.0	66.6	85.7	98.4

Table S3: Relative comparison of the lipid profile in different plant-pathogenic bacteria dependent on the number of bound BCFA for the major phospholipid classes PE, PG and CL.

PE profile [%]	*Ssc*	*Pc*	*Pst*	*Xep*	*Xcc*	*Cm*	Pool
2 BCFA	88.3	0.0	0.0	26.1	38.2	38.6	18.1
1 BCFA	11.5	0.1	2.0	50.8	45.3	49.2	14.5
no BCFA	0.2	99.9	98.0	23.2	16.5	12.3	67.3

PG profile [%]	*Ssc*	*Pc*	*Pst*	*Xep*	*Xcc*	*Cm*	Pool
2 BCFA	73.9	0.0	0.0	35.5	39.5	38.5	16.3
1 BCFA	26.1	0.1	0.8	50.1	46.4	49.5	18.6
no BCFA	0.0	99.9	99.2	14.4	14.1	12.0	65.1

CL profile [%]	*Ssc*	*Pc*	*Pst*	*Xep*	*Xcc*	*Cm*	Pool
4 BCFA	60.1	0.0	0.0	26.3	29.8	28.6	22.3
3 BCFA	30.4	0.0	0.0	39.6	40.1	44.4	20.9
2 BCFA	4.7	0.0	0.0	25.7	24.0	21.8	10.4
1 BCFA	4.4	0.1	1.0	7.3	5.1	4.0	2.7
no BCFA	0.4	99.9	99.0	1.1	1.1	1.2	43.7
